# Biomimetic Organic Nanozyme as Tumor Vaccines for Targeted Suppression of Ammonia‐Induced T Lymphocyte Death to Augment Breast Cancer Immunotherapy

**DOI:** 10.1002/advs.202518037

**Published:** 2025-11-07

**Authors:** Meng Suo, Deyi Yang, Mingpu Yang, Jue Wang, Dingfeng Zhang, Daoming Zhu, Qingyong Xu, Yanni Song

**Affiliations:** ^1^ Research Center of Nanomedicine Technology The Second Affiliated Hospital of Guangxi Medical University Nanning 530000 China; ^2^ Department of Breast Surgery Harbin Medical University Cancer Hospital 150 Haping Road Harbin 150081 China; ^3^ Department of Breast Radiotherapy Harbin Medical University Cancer Hospital Harbin Heilongjiang 150081 China; ^4^ Department of General Surgery Guangdong Provincial Key Laboratory of Precision Medicine for Gastrointestinal Tumor Nanfang Hospital Southern Medical University Guangzhou Guangdong 510515 China

**Keywords:** ammonia, immunotherapy, organic Nanozyme, T lymphocyte death, tumor vaccines

## Abstract

Ammonia‐induced T lymphocyte death (AITD) offers a new perspective on immune regulation after the activation of CD8^+^ T cells. However, the use of a single AITD inhibitor is constrained by multiple factors in the immunosuppressive tumor microenvironment and requires combination strategies to achieve breakthroughs. Herein, a rationally designed organic nanozyme (IR‐IHpd) is presented, integrating anthocyanin‐based near‐infrared photodynamic therapy (NIR‐PDT) and Hemin‐derived peroxidase (POD)‐like catalytic activity. Under 780 nm laser irradiation, it generates ROS through Type I/II photodynamic mechanisms while catalyzing H_2_O_2_ into cytotoxic ·OH, establishing an uninterrupted ROS generation. Co‐encapsulated with CB‐839 in DSPE‐Hyd‐PEG and coated with dendritic cell (DC) membranes to form a biomimetic system (DMIC), this system targets both tumors and T cells. After intravenous administration, the DMIC nanozyme system efficiently accumulates in tumor tissues, tumor‐draining lymph nodes, and spleens, where NIR irradiation induces tumor immunogenic cell death while promoting DCs maturation and T cell activation. The DMIC also functions as a tumor vaccine, capable of directly activating T cells and preventing tumor occurrence. Furthermore, the released CB‐839 reduces intracellular ammonia levels in T cells, thereby enhancing anti‐tumor immunity. This pioneering work achieves targeted AITD inhibition for the first time, integrating NIR‐PDT, metabolic modulation, and immune activation to advance nanozyme‐based immunotherapy.

## Graphical abstract

The organic nanozyme IR‐IHpd, which has NIR‐PDT and POD‐like activity, is designed for continuous ROS generation. It is co‐encapsulated with CB‐839 and coated with DC membranes to simultaneously target tumors and T cells. Under NIR irradiation, immunogenic cell death is activated. Concurrently, CB‐839 suppresses ammonia‐induced T lymphocyte death (AITD), which in turn strengthens the anti‐tumor efficacy. It pioneers targeted AITD inhibition for nanozyme‐based immunotherapy.

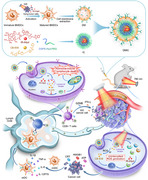

## 1 Introduction

Cancer immunotherapy currently focuses on immune checkpoint inhibitors and Chimeric Antigen Receptor T‐Cell‌‌ therapy, achieving remarkable efficacy in breast cancer, non‐small cell lung cancer, and hematological malignancies.^[^ ^1^, ^2^, ^3^, ^4^, ^5^ ^]^ However, it still faces challenges such as a “cold” tumor microenvironment and T cell exhaustion.^[^ ^6^, ^7^, ^8^ ^]^ Against this backdrop, the ammonia‐induced T lymphocyte death (AITD) offers a new perspective on immune regulation after the activation of CD8^+^ effector T cells.^[^ ^9^ ^]^ Ammonia accumulation occurs due to the absence of Carbamoyl Phosphate Synthetase 1, leading to lysosomal alkalization, mitochondrial damage, and characteristic cytoplasmic vacuolation. This mechanism not only explains the rapid contraction of T cells after antigen clearance but also provides new therapeutic targets. For instance, the ammonia scavenger 4‐phenylbutyric acid can prolong T cell survival, while the combination of ammonia metabolism regulation and CAR‐T or checkpoint inhibitors may overcome exhaustion. However, the association between AITD and the tumor microenvironment and its clinical transformation still requires in‐depth exploration. The use of a single AITD inhibitor in tumor immunotherapy faces significant limitations, as its efficacy is constrained by multiple factors and requires combination strategies to achieve breakthroughs. In the tumor microenvironment (TME), insufficient immunogenicity, impaired antigen presentation, and metabolic suppression (e.g., ammonia accumulation from glutamine metabolism leading to effector T‐cell apoptosis) collectively undermine the therapeutic effect of single inhibitors.^[^ ^10^, ^11^, ^12^, ^13^, ^14^, ^15^, ^16^, ^17^ ^]^ So far, there are no research reports on the combined targeted inhibition of AITD.

In recent years, nanomedicine has been widely studied in the treatment of tumors.^[^ ^18^, ^19^, ^20^, ^21^, ^22^, ^23^, ^24^ ^]^ Nanomaterials have demonstrated significant potential in targeting the inhibition of T‐cell AITD to sensitize tumor immunotherapy. Nanozyme materials with high catalytic activity can efficiently kill tumor cells, convert “cold” tumors into “hot” tumors, and enhance the efficacy of immunotherapy.^[^ ^25^, ^26^, ^27^, ^28^ ^]^ Nanozyme has demonstrated significant advantages in tumor treatment, including ‌ high catalytic efficiency, ‌excellent biocompatibility, and ‌ multi‐functional integration.^[^ ^5^, ^24^, ^29^, ^30^, ^31^, ^32^, ^33^, ^34^ ^]^ However, inorganic nanozyme still has potential drawbacks, such as the limitation of biocompatibility, uneven catalytic efficiency, insufficient in vivo stability, and high preparation cost. These defects limit its large‐scale application and need to be further improved through material optimization. Organic nanomaterials, such as Hemin, can self‐assemble into nanozymes with excellent anti‐tumor efficacy and superior catalytic performance, offering significant advantages over inorganic nanozymes.^[^ ^35^ ^]^ Besides inorganic and organic nanozymes, organic‐inorganic hybrid nanozymes also have great potential to be in biomedical fields, such as antibacterial, wound healing, and anti‐tumor applications.^[^ ^36^, ^37^, ^38^, ^39^, ^40^, ^41^ ^]^ However, the clinical application of single‐functional organic nanozymes remains limited, and the development of multifunctional integrated organic nanozymes represents a key future direction.^[^ ^42^ ^]^ Therefore, combining the antitumor effects of multifunctional organic nanozymes with AITD inhibitors holds promise for developing highly effective novel tumor immunotherapy.

**Scheme 1:**
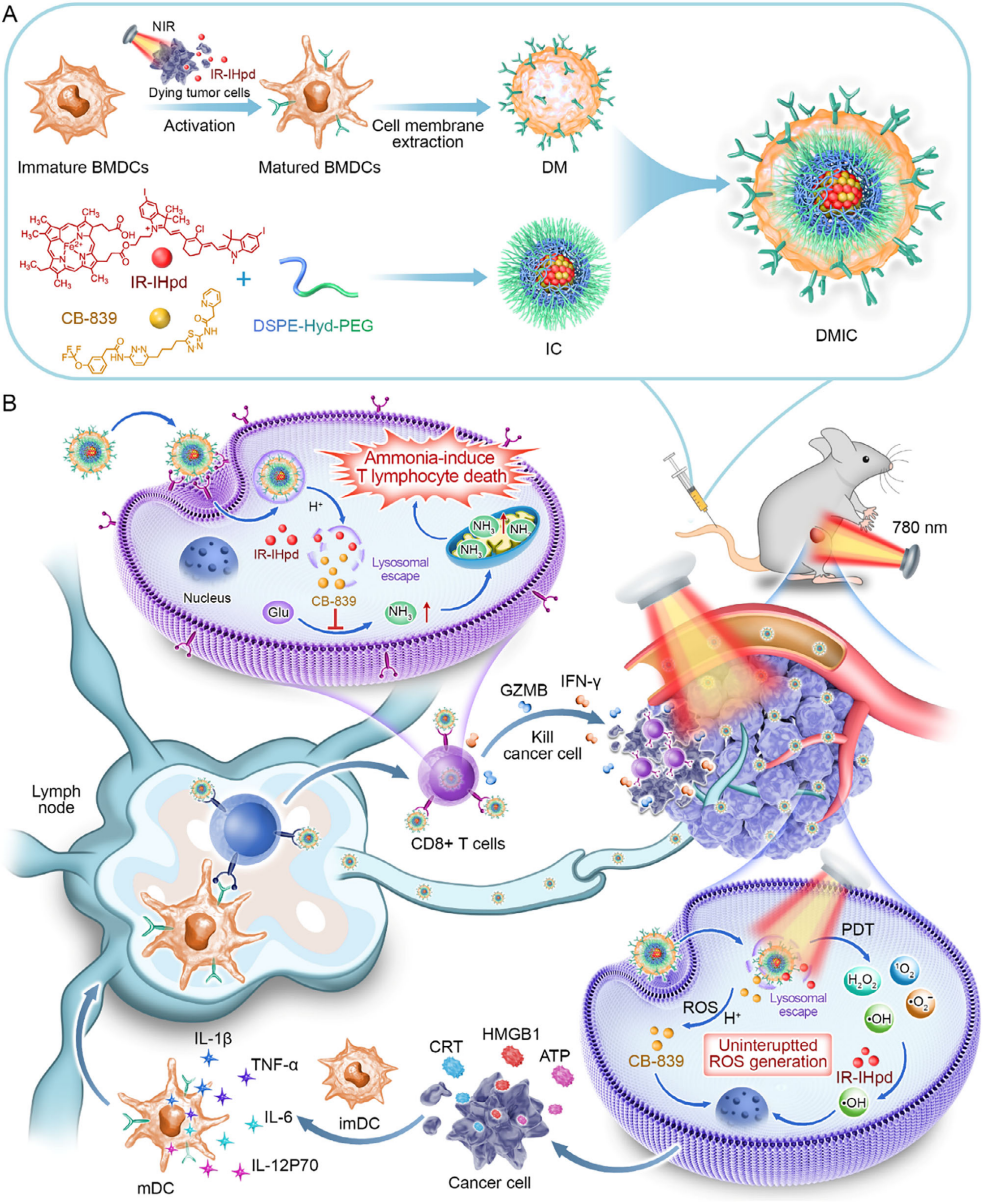
Schematic illustration of biomimetic nanozyme for targeted suppression of ammonia‐induced T lymphocyte dath to augment cancer immunotherapy. A) The preparation process of DMIC and combination therapy administration mode. B) The in vivo mechanism of DMIC plus NIR laser in anti‐tumor treatment.

Herein, we present a rationally designed organic nanozyme (IR‐IHpd) integrating anthocyanin‐based near‐infrared photodynamic therapy (NIR‐PDT) and Hemin‐derived peroxidase (POD)‐like catalytic activity (**Scheme** **1**). Under NIR laser irradiation‌, IR‐IHpd simultaneously executes Type I/II photodynamic mechanisms to generate reactive oxygen species (ROS), while its moiety ‌catalytically converts H_2_O_2_ generated by NIR‐PDT into cytotoxic hydroxyl radicals (·OH), establishing an uninterrupted ROS generation. Subsequently, we co‐encapsulated IR‐IHpd and the AITD inhibitor CB‐839 with DSPE‐Hyd‐PEG to form an acid‐responsive nanomaterial named IC. Finally, we coated IC with mature bone marrow‐derived dendritic cells (BMDCs) cell membranes (DM) to form biomimetic DMIC nanoparticles. Cell membrane‐mimicking nanomaterials, as a cutting‐edge technology in the biomedical field, have achieved remarkable breakthroughs in recent years in areas such as cancer treatment, drug delivery for brain diseases, and antibacterial applications.^[^ ^43^ ^]^ Tumor cell membrane‐mimicking nanoparticles significantly enhance the drug accumulation at tumor sites through homologous targeting characteristics, while also achieving immune evasion through CD47 protein and prolonging the circulation time in the body.^[^ ^18^, ^44^, ^45^ ^]^ Mature BMDCs highly express key immunomodulatory proteins, including MHC‐I, CD80, CD86, and tumor‐associated antigens, enabling them to simultaneously target T cells and tumor cells in lymph nodes and tumor tissues, making their membranes ideal carriers for addressing AITD.^[^ ^46^, ^47^ ^]^ DSPE‐Hyd‐PEG is a pH‐responsive phospholipid material that can efficiently encapsulate hydrophobic drugs and rapidly release them under acidic conditions.^[^ ^48^, ^49^ ^]^ Our experimental results demonstrate that after intravenous administration, the DMIC nanozyme efficiently accumulates in tumor tissues, tumor‐draining lymph nodes, and spleens. Upon near‐infrared laser irradiation, DMIC generates a surge of ROS within tumors, triggering immunogenic cell death (ICD) in tumor cells while promoting dendritic cell (DC) maturation and T cell activation. The DMIC also functions as a tumor vaccine, capable of directly activating T cells and preventing the occurrence of tumors. Furthermore, upon internalization by T cells, DMIC releases CB‐839, a potent inhibitor of AITD, which reduces intracellular ammonia levels and ROS production in T cells, thereby enhancing anti‐tumor immunity. This study pioneers the development of an innovative organic nanozyme system that achieves selective inhibition of AITD for the first time. By integrating targeted T cell drug delivery, ICD induction, and metabolic modulation, DMIC provides groundbreaking insights for the rational development of next‐generation anti‐tumor nanozymes and advanced immunotherapeutic models.

## 2 Results and Discussion

### 2.1 Preparation and Characterization of DMIC

**Figure 1:**
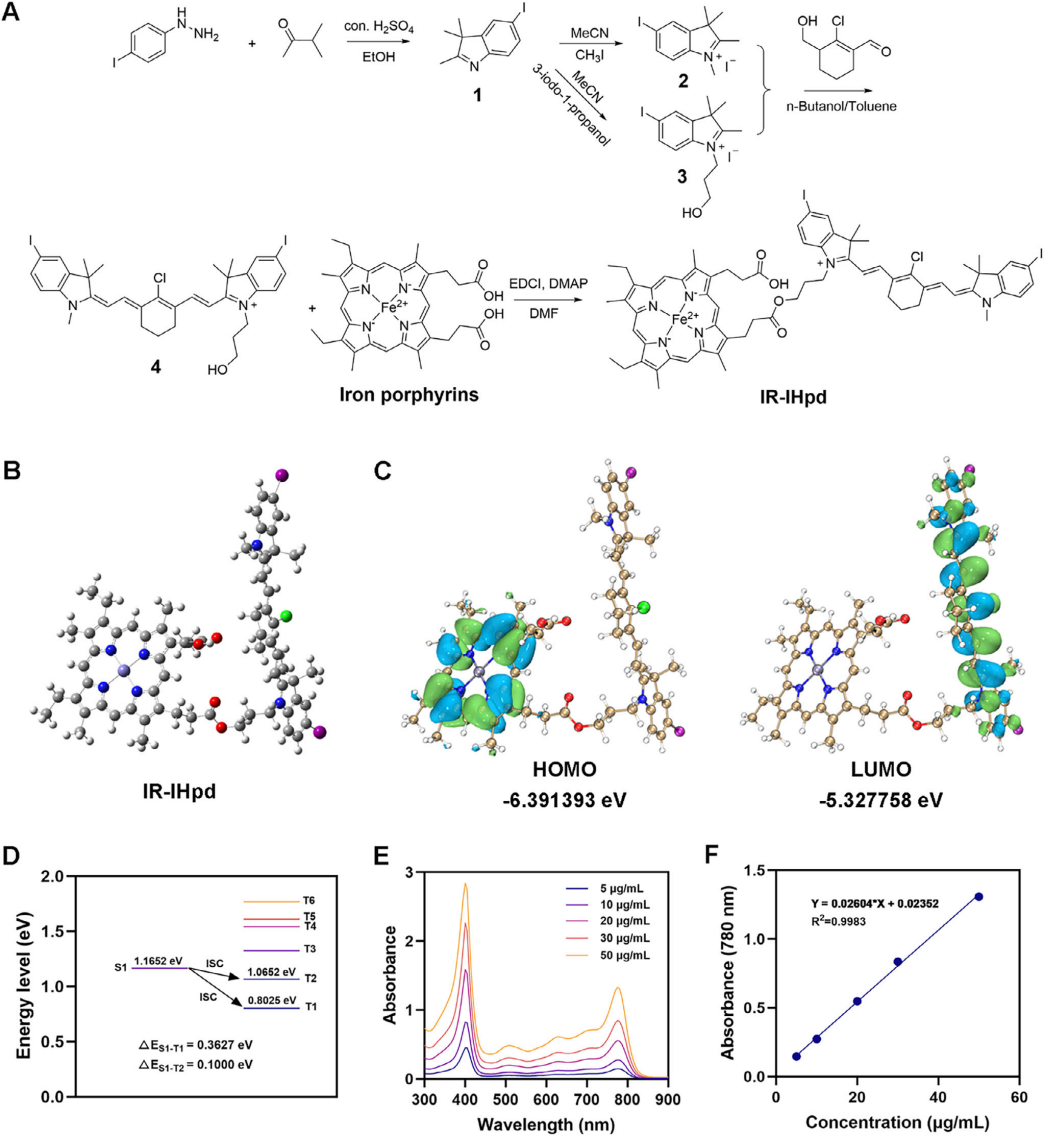
A) Synthetic route of IR‐IHpd. B) 3D chemical structure of IR‐IHpd. C) HOMO‐LUMO distribution determined by DFT calculations for IR‐IHpd. D) Energy levels for simplified IR‐IHpd at the optimized molecular geometry. E) Absorption spectra of IR‐IHpd in DMSO solution at different concentrations. F) Standard Curve of IR‐IHpd.

We successfully synthesized a novel small molecule cyanine photosensitizer, designated as IR‐IHpd. The synthetic route, molecular structure, and comprehensive characterization data, including High‐Resolution Mass Spectrometry (HRMS) and ^1^H Nuclear Magnetic Resonance (NMR) spectra for both IR‐IHpd and its intermediates are detailed in **Figure** **1**A and Figures S1–S6 (Supporting Information), unequivocally confirming the successful synthesis of these compounds. To investigate the triplet‐state formation mechanism, we performed theoretical studies on IR‐IHpd using time‐dependent density functional theory (TD‐DFT). As shown in Figure 1B,C, DFT calculations determined the Lowest Unoccupied Molecular Orbital (LUMO) and Highest Occupied Molecular Orbital (HOMO) energy levels of IR‐IHpd to be −5.328 and −6.391 eV, respectively. The calculated bandgap (Eg) of 1.064 eV suggests a narrow energy gap, significantly enhancing its near‐infrared (NIR) light absorption properties.^[^ ^50^, ^51^ ^]^ Notably, the energy differences (ΔE_S1−T1_ = 0.363 eV and ΔE_S1−T2_ = 0.100 eV) in Figure 1D indicate efficient intersystem crossing (ISC) pathways from the S1 state to both T1 and T2 states, which is critical for boosting ROS generation.^[^ ^51^, ^52^ ^]^ Furthermore, the optical absorption spectrum and corresponding standard curve at 780 nm (Figure 1E,F) corroborate the compound's strong NIR absorption capability.

**Figure 2:**
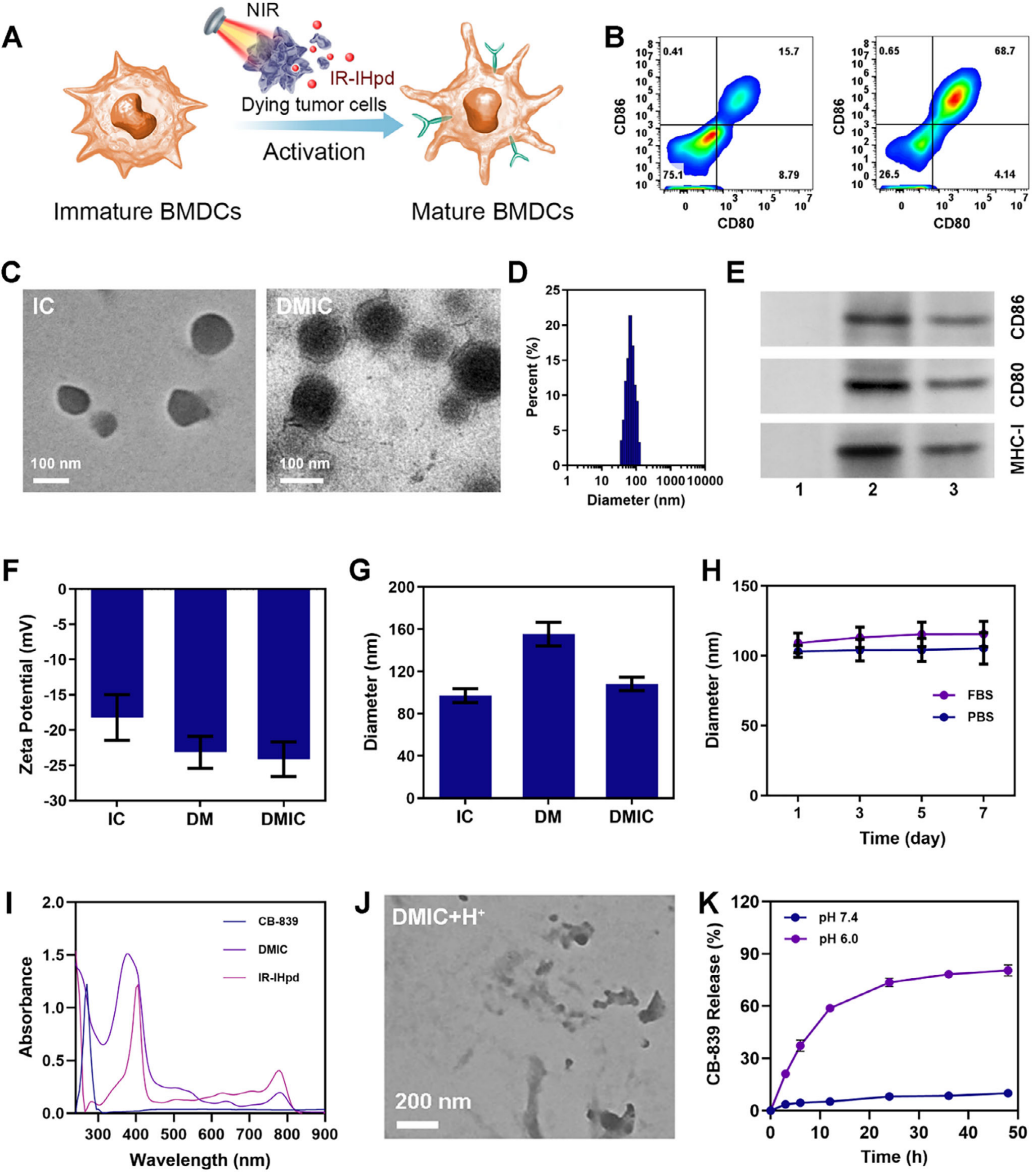
A) BMDCs activation process. B) Flow cytometry (FCM) analyses of BMDCs labeled with anti‐CD80 and CD86 antibodies after co‐culture with tumor cell lysate (TCL). C) TEM image of IC and DMIC. D) The particle size distribution of DMIC. E) Western blot (WB) analysis of key proteins in different formulations (1: IC, 2: DM, 3: DMIC). F) Zeta potential and G) Particle diameter measurements for IC, DM, and DMIC. H) Stability of DMIC in various solutions. I) Absorption spectra of indicated formulations. J) TEM image of DMIC after H^+^ treatment (pH = 6) for 4 h. K) Drug release curves of DMIC under different pH conditions. Data are shown as the mean ± SD (*N* = 3).

The fabrication of DMIC followed a sequential protocol. First, IR‐IHpd and CB‐839 were co‐encapsulated with DSPE‐Hyd‐PEG2000 to assemble the core nanoparticle (designated as IC). Next, mature BMDCs membranes (DM) were physically extruded onto the IC surface to yield the final DMIC nanocomposite (**Figure** **2**A). For DM preparation, IR‐IHPD‐treated 4T1 cells underwent laser‐induced photoactivation to produce tumor cell lysates (TCL), which were then co‐cultured with immature BMDCs to promote BMDCs maturation, as evidenced by flow cytometric analysis of surface markers (Figure 2B). TEM characterization clearly visualized the membrane coating on DMIC's surface (Figure 2C,D, Figure S7, Supporting Information), showing a particle size increase of ≈10 nm compared to IC and a zeta potential profile matching DM (Figure 2D,F,G), confirming successful membrane encapsulation. Western blot assay verified the retention of characteristic DM markers (CD80, CD86, and MHC‐I) on DMIC (Figure 2E, Figure S8, Supporting Information). The nanocomposite maintained superior stability (Figure 2H). Quantitative analysis using absorption spectroscopy revealed drug loading efficiencies of 42.4 ± 3.2% for IR‐IHpd and 33.5 ± 2.1% for CB‐839 (Figure 2I). Importantly, DMIC displayed pH‐triggered drug release kinetics, with rapid CB‐839 liberation under acidic conditions (Figure 2J,K).

**Figure 3:**
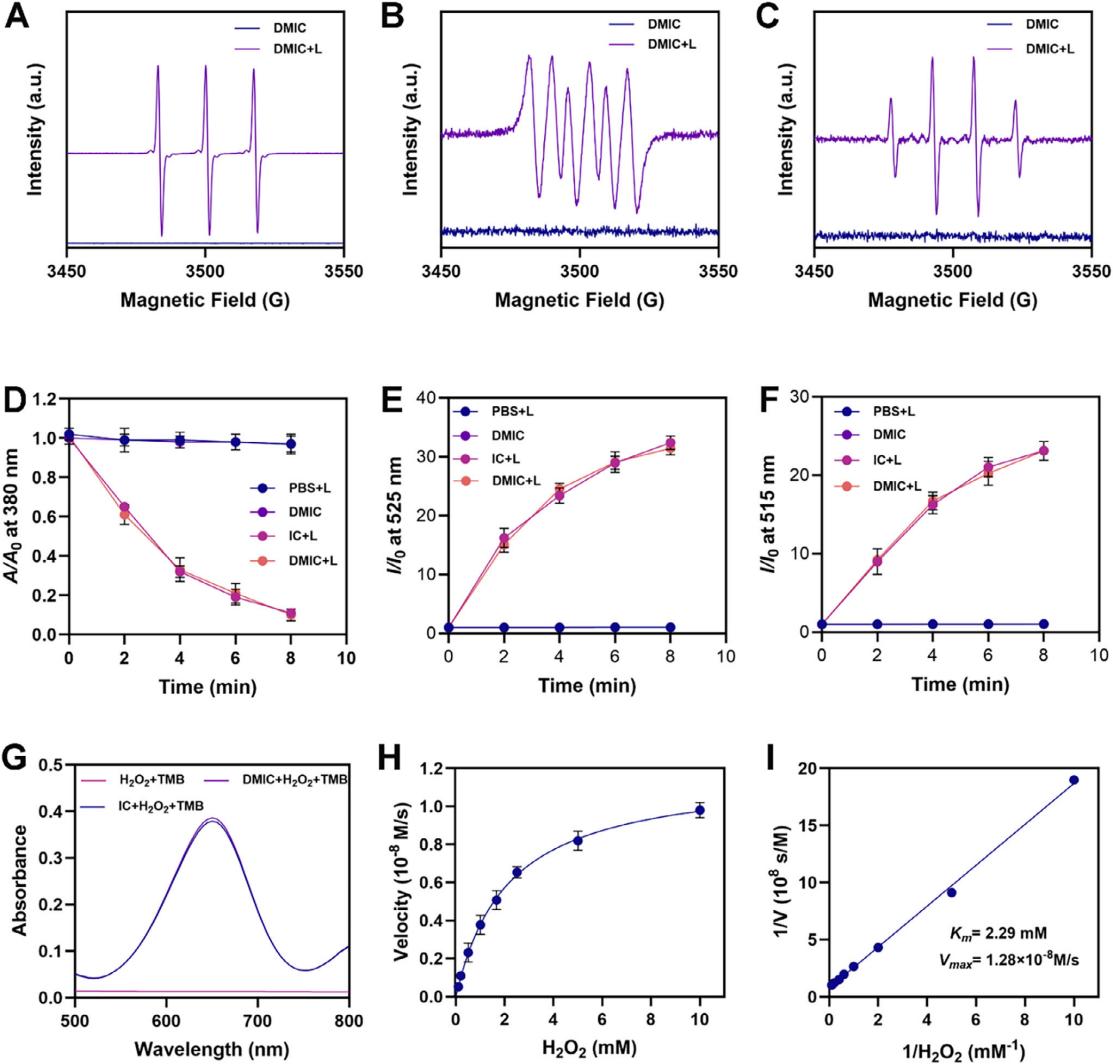
A) Production of ^1^O_2_, B) ·O_2_^−^, and C) •OH by the DMIC or DMIC+L (780 nm, 0.5 W cm^−2^, 3 min) was determined by ESR. D) ABDA decomposition of different formulations. E) Relative changes in the fluorescence of DHR123 and F) HPF. G) Absorbance changes of TMB due to the generated •OH by indicated treatments. H) Initial velocity of the reaction with a varied concentration of H_2_O_2_. I) The double‐reciprocal plots of H_2_O_2_. Data are shown as the mean ± SD (*N* = 3).

Electron spin resonance (ESR) spectroscopy was employed to investigate ROS generation in both IR‐IHpd and DMIC. The experimental data presented in **Figure** **3**A–C demonstrate that DMIC is capable of simultaneously producing singlet oxygen (^1^O_2_), hydroxyl radicals (·OH), and superoxide anions (·O_2_^−^) upon 780 nm laser irradiation. Time‐dependent ROS production by DMIC was systematically evaluated using specific indicators, as illustrated in Figure 3D–F. These observations conclusively establish DMIC's dual functionality in performing both type I and type II photodynamic therapy (PDT) under NIR laser excitation. The ability of type I PDT photosensitizers to effectively overcome  hypoxic tumor microenvironments, as supported by prior research, underscores DMIC's significant therapeutic potential.^[^ ^19^, ^53^, ^54^, ^55^ ^]^ To further characterize DMIC's peroxidase‐like activity (POD), we employed tetramethylbenzidine (TMB) as a detection probe for ·OH generation during Fenton‐type reactions. The oxidation process resulted in a distinct color transition from colorless to bluish green, accompanied by the emergence of an absorption peak at 652 nm.^[^ ^56^ ^]^ These results provide definitive evidence that both IC and DMIC, when encapsulated within IR‐IHpd, can effectively catalyze hydrogen peroxide (H_2_O_2_) to produce ·OH, as quantitatively shown in Figure 3G. A comprehensive kinetic characterization of DMIC's nanozymatic activity was systematically performed through Lineweaver‐Burk analysis.^[^ ^25^, ^57^, ^58^ ^]^ The obtained Michaelis‐Menten parameters (Figure 3H,I) indicate a *K_m_* value of 2.29 mM and a *V_max_* of 1.28 × 10^−8^ M/s for H_2_O_2_ decomposition. These results unequivocally confirm the successful development of DMIC as a multifunctional platform, integrating outstanding NIR absorbance, potent ROS generation capacity, and superior chemical stability. All critical attributes that fulfill the fundamental requirements for forthcoming in vivo biological studies.

### 2.2 In Vitro Anti‐Tumor Ability of DMIC

**Figure 4:**
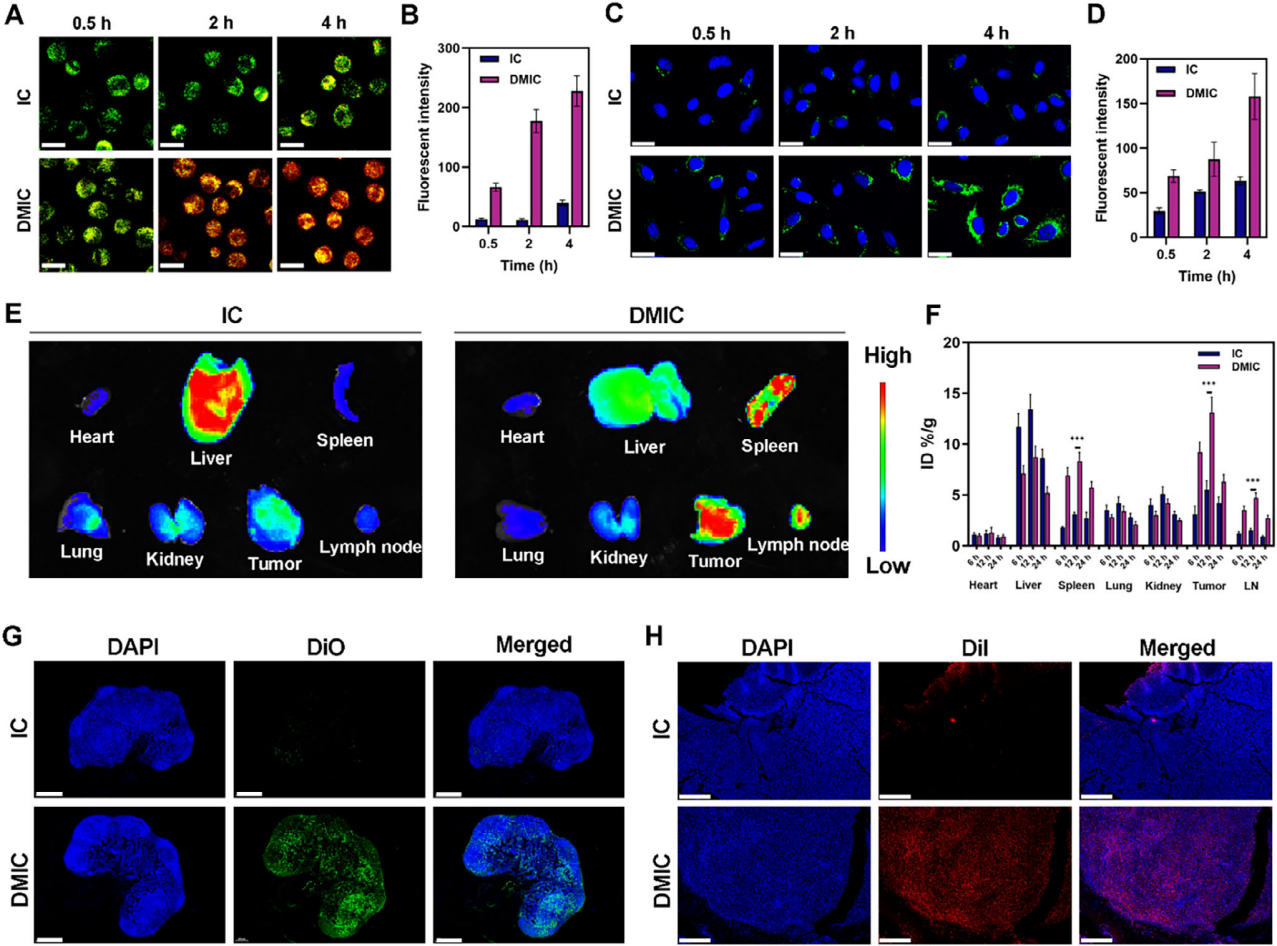
A) Confocal laser scanning microscopy (CLSM) images and B) quantitative red fluorescence intensity of CD8^+^ T cells treated with Dil‐labeled IC or DMIC. Scale bars: 10 µm. Green: Lyso‐tracker Green, Red: Dil. (C) CLSM images and D) quantitative green fluorescence intensity of 4T1 cells treated with DiO‐labeled IC or DMIC. Green: DiO, Blue: DAPI. Scale bars: 20 µm. E) Ex vivo fluorescence imaging of tumors, tumor‐draining lymph nodes, and organs from 4T1 tumor‐bearing mice after injection of IC or DMIC for 12 h. F) Quantitative in vivo biodistribution analysis of CB‐839 in different formulations at 6, 12, and 24 h after intravenous injection in mice bearing 4T1 breast tumor. Data are shown as the mean ± SD (*N* = 3). G) Immunofluorescence images of LNs treated with DiO‐labeled IC or DMIC after 12 h injection. Scale bars: 400 µm. H) Immunofluorescence images of tumors treated with Dil‐labeled IC or DMIC after 12 h injection. Scale bars: 400 µm. Data are shown as the mean ± SD (*N* = 3). Statistical significance was calculated via one‐way ANOVA with Tukey's test: ^***^ *p* < 0.001.

Given the favorable physicochemical properties of DMIC, we proceeded to evaluate its in vitro anti‐tumor efficacy. Accumulating evidence suggests that the immune cells' membrane can confer nanoparticles with enhanced biocompatibility and immune evasion capabilities, facilitate membrane‐specific molecular recognition, and enable targeted delivery to both immune and tumor cells.^[^ ^43^, ^59^ ^]^ To determine whether DMIC could interact with T cells, we co‐incubated DMIC with T cells in vitro and employed Lysotracker to label the T cell membrane (green fluorescence), while Dil was used to label the nanomaterials as a red fluorescent marker for both IC and DMIC. Confocal laser scanning microscope results revealed that, compared to IC, DMIC exhibited significantly enhanced binding to the surface of T cells after an equivalent incubation period (**Figure** **4**A,B). This improved binding capacity may be attributed to the functional loading of the DM, which facilitates more efficient T cell targeting and activation by DMIC. In addition, after incubation for more than 2 h, DMIC showed obvious lysosomal escape, which might be due to the formation of aggregates by the released drugs.^[^ ^60^ ^]^ To further assess the tumor‐targeting capability of DMIC, it was co‐cultured with 4T1 tumor cells. As shown in Figure 4C,D, after 4 h of incubation, DMIC demonstrated an increased tumor cell enrichment compared to IC, suggesting superior internalization by tumor cells. Collectively, these findings demonstrate that DMIC can effectively target both T cells and tumor cells. T cells may serve as carriers to transport DMIC across vascular barriers and into the tumor microenvironment, thereby further enhancing nanoparticle delivery efficiency. To validate the lymph node homing and tumor‐targeting abilities of this biomimetic artificial DC, we compared IC and DMIC by measuring ex vivo organ fluorescence intensity 24 h after tail vein injection in mice. As illustrated in Figure 4E, ex vivo imaging revealed significantly higher fluorescence signals in the spleen, tumor tissue, and lymph nodes of the DMIC group. Pharmacokinetic and biodistribution experiments demonstrated that DMIC has better blood long‐circulation ability (Figure S9, Supporting Information), and the maximum tumor accumulation was reached 12 h after intravenous injection (Figure 4F). Immunofluorescence staining of tumor and lymph node tissues further confirmed greater DMIC accumulation, supporting its role as an artificial antigen‐presenting cell with in vivo tumor and lymph node targeting capacity (Figure 4G,H). These results collectively indicate that DMIC not only migrates to lymph nodes but also effectively adheres to T cells, thereby being transported to the tumor microenvironment and enhancing tumor targeting.

**Figure 5:**
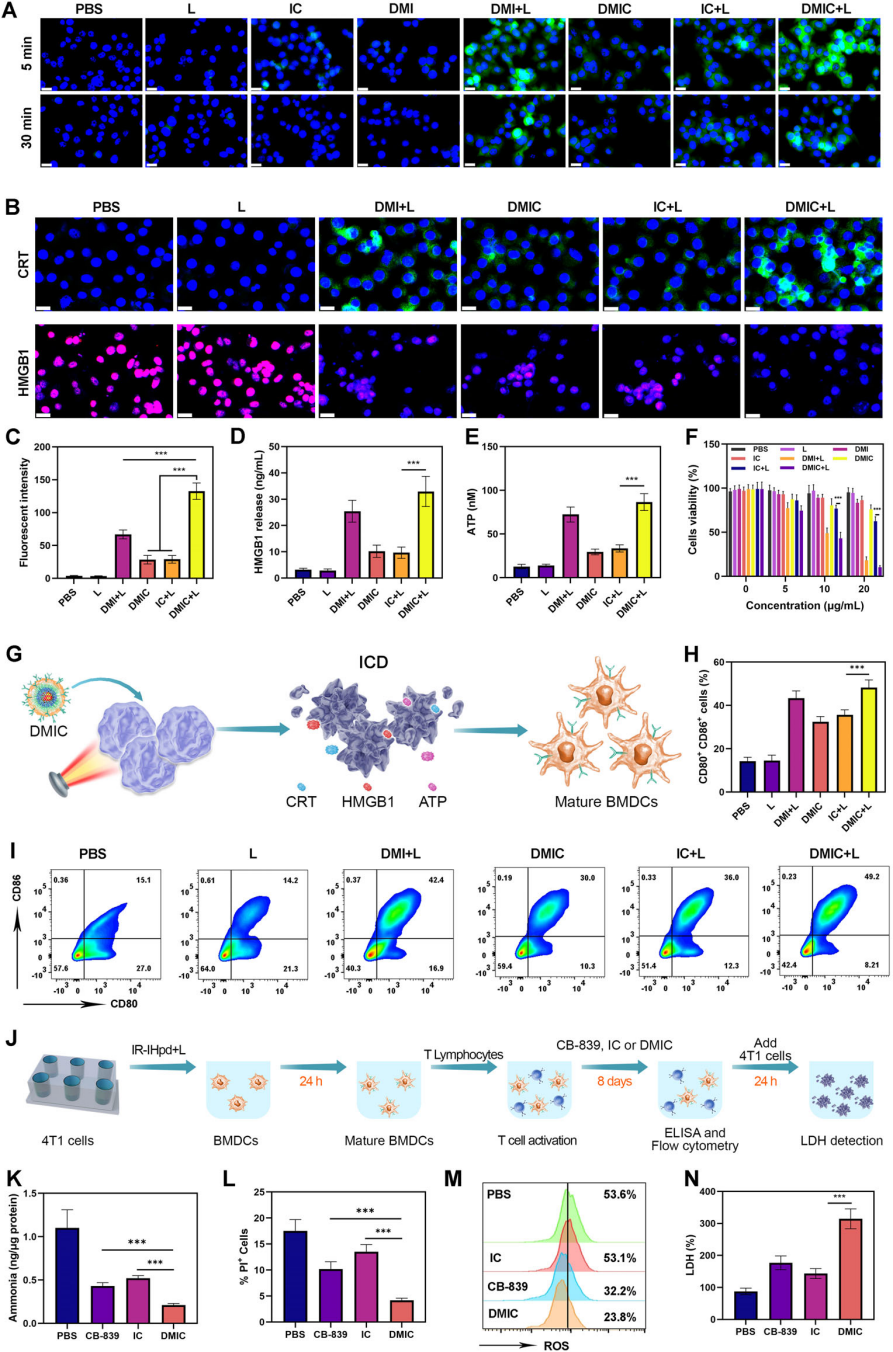
A) CLSM images and of •OH (green fluorescence) generated in 4T1 cells upon different times after treatments. Scale bars: 20 µm. B) •OH fluorescence intensity of Figure 4A (30 min). C) Expression of HMGB1 and CRT (scale bars: 20 µm). D) CRT fluorescence intensity of Figure 4C. E) HMGB1 and F) ATP release from 4T1 cells following treatment with different formulations (L: 780 nm laser, 0.5 W cm^−2^, 5 min). F) The cell viability of 4T1 cells after various treatments with different IR‐IHpd concentrations. G) Schematic illustration of the Transwell system used to induce maturation of BMDCs in vitro. H) Quantitative analysis and I) Flow cytometry analysis of mature BMDCs after different treatments. J) A schematic representing specific immune activation experimental setups in vitro. K) Ammonia levels, L) propidium iodide staining, and M) intracellular ROS levels were measured 10 days after T cell activation. N) Measurement of LDH levels in the supernatant after co‐incubation of the T lymphocytes treated by the indicated treatment with 4T1 tumor cells for 24 h. Data are shown as the mean ± SD (*N* = 3). Statistical significance was calculated via one‐way ANOVA with Tukey's test: ^***^ *p* < 0.001.

After confirming ROS generation mediated by DMIC under 780 nm laser irradiation in aqueous solution, we further assessed intracellular ROS production in 4T1 tumor cells. Type‐I PDT was reported to elicit H_2_O_2_ generation,^[^ ^54^ ^]^ which agrees with our research findings (Figure S10, Supporting Information) and provides the prerequisite for abundant intracellular ·OH generation by DMIC. The HPF probe was employed to detect ·OH generation. As shown in **Figure** **5**A, bright green fluorescence was clearly observed in the DMI+L and DMIC+L treatment groups upon laser irradiation. Moreover, the levels of ROS generated in cells treated with DMI+L or DMIC+L were significantly higher than those in the IC+L group, likely due to the biomimetic cell membrane‐mediated targeting that enhanced tumor cell uptake of DMIC+L (or DMI+L). In contrast, only weak green fluorescence was detected in the IC+L control group. Notably, even after 30 min of irradiation, substantial ROS production was still observed in the DMI+L and DMIC+L groups, indicating that these treatments can induce sustained ROS generation, thereby enhancing cytotoxicity against tumor cells. Efficient ROS generation can trigger immunogenic cell death (ICD), leading to the release of damage‐associated molecular patterns (DAMPs), such as calreticulin (CRT) and high mobility group box 1 (HMGB1). As expected, immunofluorescence analysis confirmed that CRT surface exposure was significantly increased in the DMI+L and DMIC+L groups compared to other control groups, while nuclear HMGB1 levels were markedly reduced (Figure 5B,C). In the DMIC+L group, nearly all nuclear HMGB1 was translocated to the extracellular space, and Adenosine Triphosphate (ATP) release was significantly elevated, providing strong evidence for ICD induction by DMI+L or DMIC+L (Figure 5D,E). Next, the in vitro cytotoxicity of nanoparticles against 4T1 cells at varying IR‐IHpd concentrations was evaluated using a cell viability assay (Figure 5F). Under laser irradiation, the cell survival rates in the DMI+L and DMIC+L groups were significantly lower than those in other control groups, with the DMIC+L group showing the most pronounced cytotoxic effect in a concentration‐dependent manner. These results indicate that DMI+L and DMIC+L can effectively induce tumor cell death in vitro. Following confirmation of ICD induction by DMI+L or DMIC+L, we further evaluated their DCs activation potential (Figure 5G). 4T1 cells were co‐cultured with immature bone marrow‐derived dendritic cells (BMDCs), and the expression of mature DC markers CD80 and CD86 was analyzed by flow cytometry. Compared with the untreated control group, both DMIC and IC+L treatments promoted BMDC maturation. Notably, DMI+L or DMIC+L treatment further enhanced BMDC maturation (Figure 5H,I). This was corroborated by increased secretion of Tumor Necrosis Factor‐alpha (TNF‐α), Interferon‐gamma (IFN‐γ), and Interleukin‐6 (IL‐6), as measured by Enzyme‐Linked ImmunoSorbent Assay (ELISA) (Figures S11–S13, Supporting Information). Cells utilize ATP as an energy source during metabolic processes. During ATP production, T cells generate ammonia (NH_3_) and ROS as by‐products, both of which are potentially cytotoxic and may limit cell viability and lifespan. Previous studies have demonstrated that intracellular ammonia accumulates progressively during CD8^+^ T cell activation, which can impair T cell growth and function, ultimately leading to T cell exhaustion and resistance to immunotherapeutic interventions. Therefore, reducing intracellular ammonia levels and scavenging ROS may support the formation and long‐term maintenance of CD8+ T cells, thereby enhancing the efficacy of T cell‐based anti‐tumor immunotherapy. To further investigate the immunomodulatory potential of DMIC, we established a ternary co‐culture system comprising BMDCs, T lymphocytes, and 4T1 tumor cells (Figure 5J). Following antigen stimulation, CD8^+^ T cells were activated and entered the clonal expansion phase. Subsequently, T cell ammonia levels were assessed after various treatments (CB‐839, IC, or DMIC). We observed a significant increase in ammonia levels in the PBS‐treated control group (Figure 5K). Recent studies revealed that rhesus family glycoproteins (RHAG, RHBG, and RHCG) can function as ammonia transporters.^[^ ^9^, ^61^ ^]^ Notably, increased RhCG appeared in late‐activated CD8^+^ T cells (Figure S14, Supporting Information), concomitant with increased ammonia levels in the CD8^+^ T cells. Flow cytometric analysis further revealed substantial T cell apoptosis in this group. In contrast, DMIC treatment significantly reduced intracellular ammonia concentrations and markedly decreased T cell apoptosis, indicating a strong correlation between ammonia accumulation and T cell death (Figure 5L). These findings suggest that DMIC effectively lowers ammonia levels and promotes T cell survival. We further evaluated ROS production in T cells under different treatment conditions (Figure 5M). Flow cytometric analysis demonstrated that ROS levels in CD8^+^ T cells were reduced following DMIC treatment compared to the control group, indicating that DMIC enhances the antioxidant capacity of T cells. Studies have shown that the accumulation of mitochondrial ROS induced by ammonia eventually leads to T cell death.^[^ ^9^ ^]^ Efficient and timely ROS clearance contributes to improved T cell functionality and persistence.^[^ ^62^ ^]^ To further assess whether DMIC can activate antigen‐specific T cells and mitigate T cell exhaustion, we evaluated the cytotoxic activity of T cells against tumor cells in vitro. Lactate dehydrogenase release assays revealed that the tumor‐killing efficiency of T cells in the DMIC group was significantly higher than that in the control groups (Figure 5N). Collectively, these results confirm that DMIC treatment effectively reduces ammonia levels, decreases T cell apoptosis, suppresses ROS accumulation, and upregulates the expression of cytotoxic molecules such as granzyme B. DMIC not only presents antigens to activate specific CD8^+^ T cell subsets but also enhances the function of exhausted T cells by modulating metabolic stressors such as ammonia and ROS.

**Figure 6:**
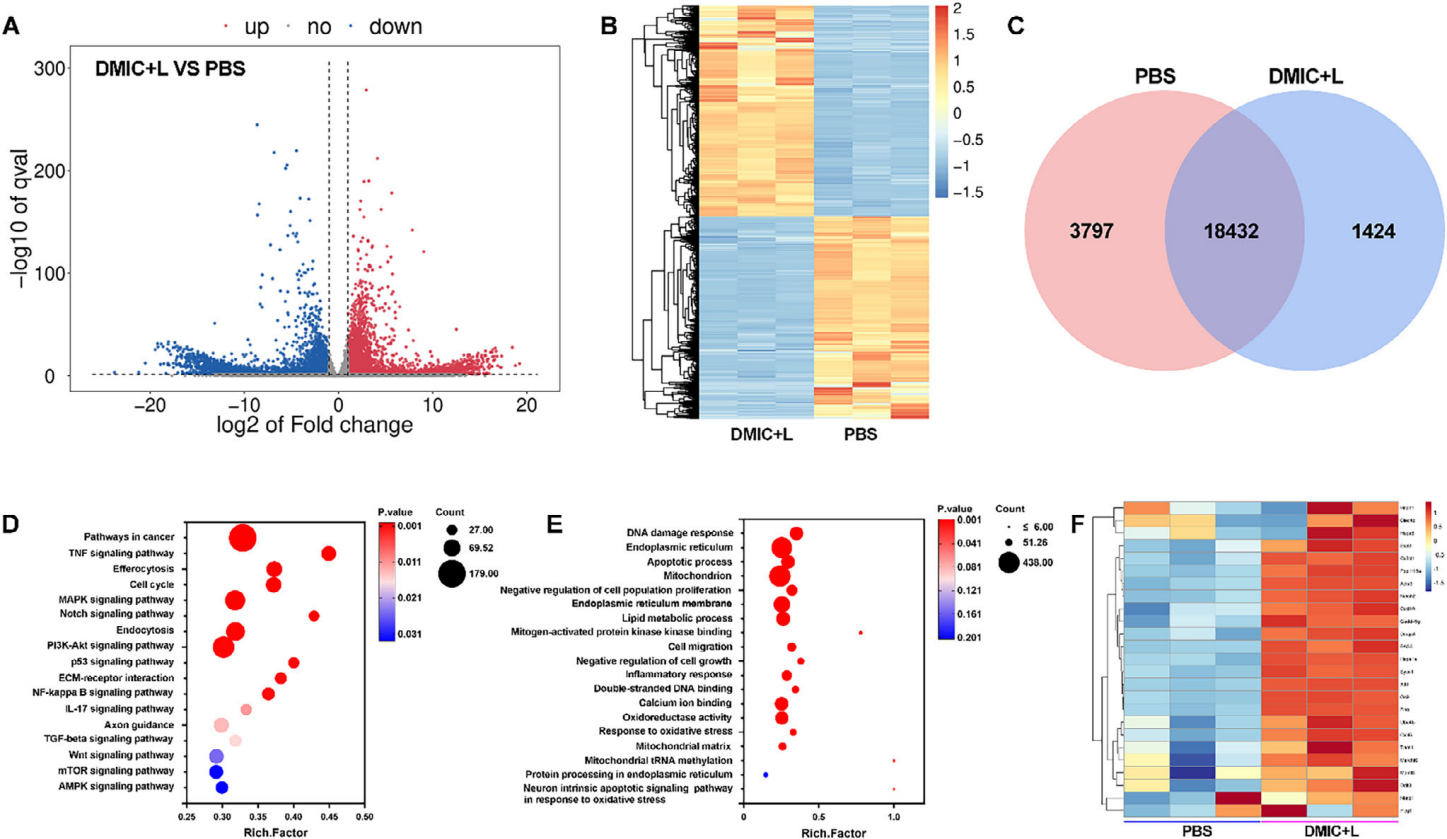
A) Volcano plots illustrating differentially expressed genes. B) Heatmap illustrating differentially expressed genes after treatment. C) The Venn diagram revealed the number of genes transcribed in the indicated treatment group. D) Kyoto Encyclopedia of genes and genomes (KEGG) enrichment and E) Gene Ontology (GO) enrichment analysis. F) Heatmap of gene expressions related to apoptosis, immunity, and oxidative stress in 4T1 cells treated with PBS and DMIC+L. L: 780 nm laser, 0.5 W cm^−2^, 5 min. E) Results of KEGG and (F) GO pathway enrichment analysis.

The results of transcriptomic sequencing illustrated that differential genes were significantly enriched in signaling pathways related to ICD, ROS, and immune regulation, further proving the potential of DMIC in immunotherapy (**Figure** **6**A–F). Relative to the PBS group, DMIC+L treatment upregulated 3044 genes and 5228 transcripts while downregulating 2932 genes and 6085 transcripts. For instance, the transcription levels of ATF3 and HSPA1A, related to ROS‐induced endoplasmic reticulum stress,^[^ ^63^, ^64^ ^]^ were upregulated, likely due to ICD. Elevated transcription levels of GADD45G, NOTCH2, and BAG3 linked to apoptosis,^[^ ^65^ ^]^ indicated profound apoptosis triggered by DMIC+L. Further Kyoto Encyclopedia of genes and genomes (KEGG) enrichment and Gene Ontology (GO) enrichment analysis revealed that genes related to protein processing in endoplasmic reticulum, apoptosis, DNA damage, mitochondrial damage, and oxidative stress, etc, were greatly affected by DMIC+L.

### 2.3 In Vivo Anti‐Tumor Ability of DMIC

**Figure 7:**
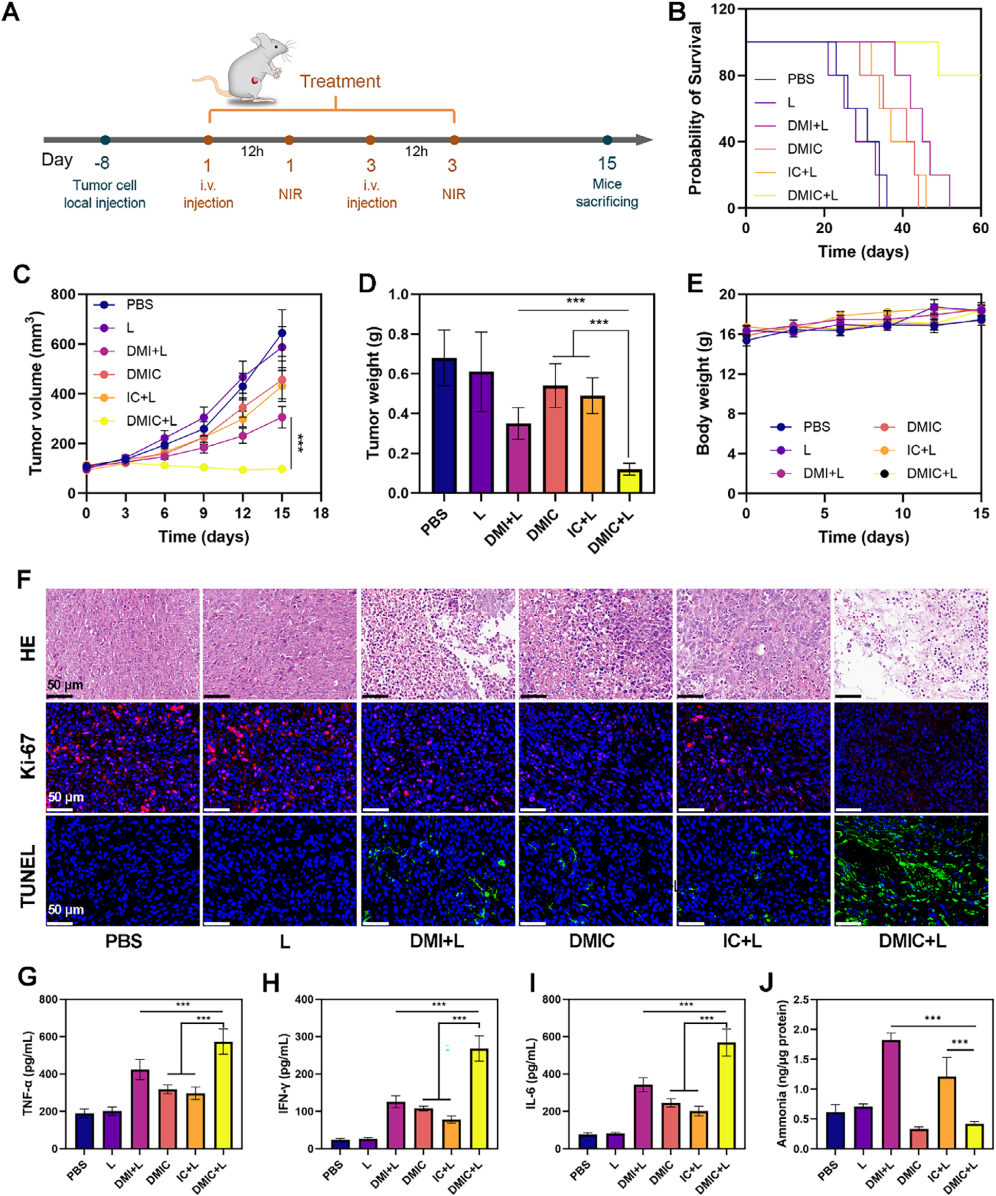
A) A schematic diagram outlining of the timeline for tumor inoculation, nanodrug administration, laser treatment, and monitoring of tumor growth. On the 15th day, mice were sacrificed. Tumors, organs, and LNs were collected for subsequent experiments. Another group of mice repeated the experiment, and their survival was observed for 60 days. B) Survival curves after treatment. C) Tumor volumes were measured every 3 days across all groups. D) Tumor weights were recorded for each treatment at the end of the study. E) Body weights were recorded for each treatment at the end of the study of bilateral murine tumor models (*N* = 5). F) HE, TUNEL, and Ki‐67 staining of tumor sections after the indicated treatments. Scale bars: 20 µm. G) Secretion of pro‐inflammatory cytokines TNF‐α, H) IFN‐γ, and I) IL‐6 in sera after exposure to different treatments. J) Ammonia levels within CD8^+^ T cells were measured 10 days after the first treatment. IR‐IHpd dose: 10 mg kg^−1^. Data are shown as the mean ± SD (*N* = 5). Statistical significance was calculated via one‐way ANOVA with Tukey's test: ^***^ *p* < 0.001.

**Figure 8:**
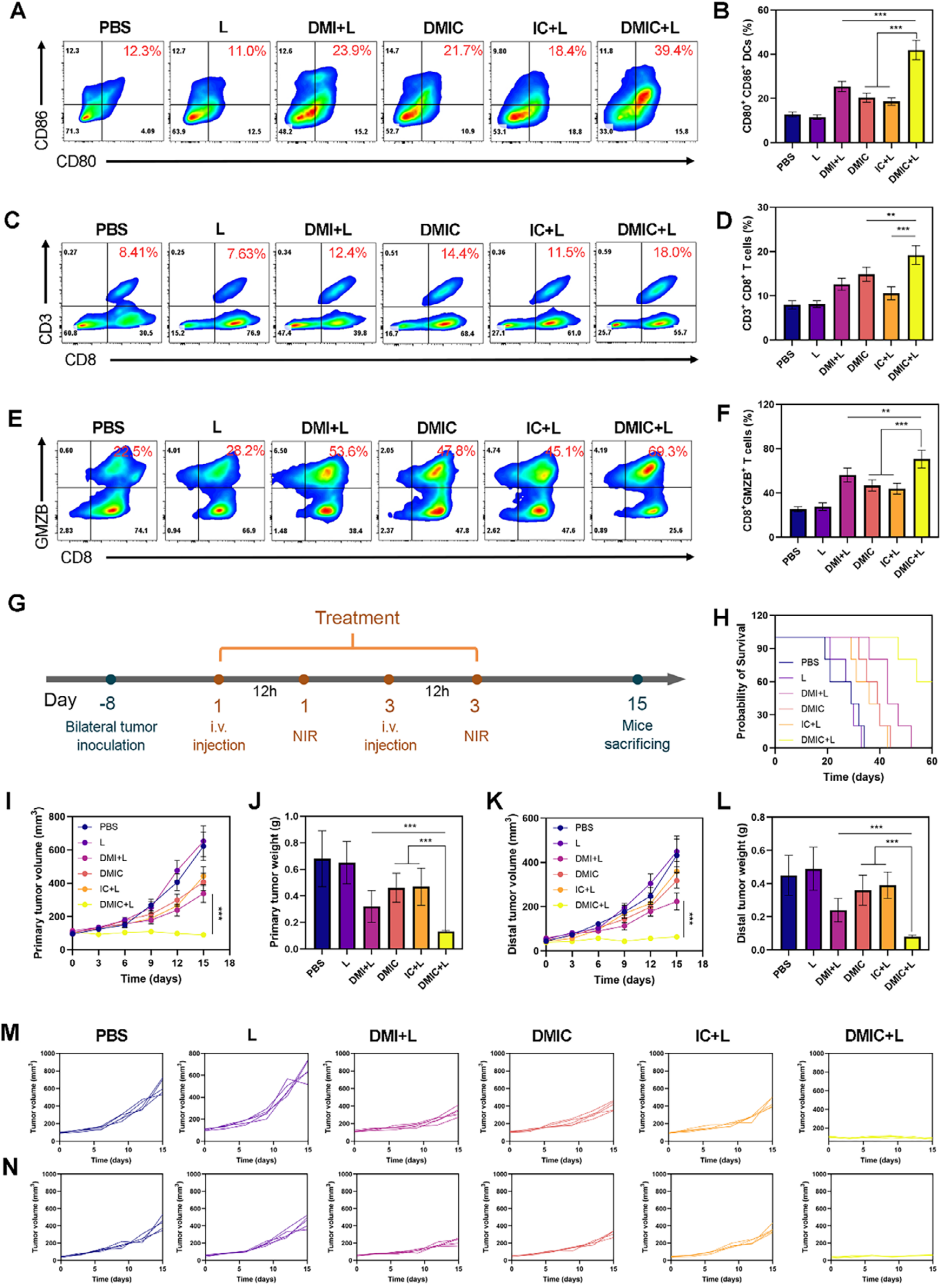
A) Flow cytometry data and B) quantitative analysis showing the effects of treatments on DCs maturation in lymph nodes on the 15th day. C) Flow cytometry data and D) quantitative analysis showing the CD3^+^ CD8^+^ T lymphocytes cells in lymph nodes on the 15th day. E) Flow cytometry data and F) quantitative analysis showing the GZMB^+^ CD8^+^ T cells in LNs on the 15th day. G) Schematic illustration of the studies of 4T1 bilateral tumor therapy. H) Survival curves after different treatments. I) Evolution of the primary tumor volume and J) tumor weight after various treatments. K) Development of distant tumor volume and L) tumor weight following various treatments. M) Primary tumor and N) distant tumor growth curve of each mouse in different groups. Data are shown as the mean ± SD (*N* = 5). Statistical significance was calculated via one‐way ANOVA with Tukey's test: ^**^ *p* < 0.01, ^***^ *p* < 0.001.

Building upon the encouraging in vitro results, we extended our investigation to evaluate the therapeutic efficacy of DMIC‐mediated near‐infrared (NIR) photoimmunotherapy in an in vivo tumor model. The experimental protocol, illustrated in **Figure** **7**A, involved intravenous administration of DMIC via the tail vein on day 0 and day 3, followed by photodynamic therapy (PDT) 12 h post‐injection. Comprehensive therapeutic outcomes were assessed, including survival analysis (Figure 7B), longitudinal tumor volume monitoring (Figure 7C), and final tumor weight quantification at day 18 (Figure 7D–E). The experimental results unequivocally demonstrate that DMIC+L treatment exhibits the most pronounced anti‐tumor efficacy and survival benefits among the tested regimens. Comprehensive histological analyses, including hematoxylin‐eosin (HE) staining and immunofluorescence‐based TUNEL assays, revealed extensive tumor necrosis and apoptotic cell death in DMIC+L‐treated specimens (Figure 7F). The DMIC+L treatment regimen demonstrated profound anti‐tumor activity through dual mechanisms: 1) marked suppression of cellular proliferation, as quantified by significantly reduced Ki‐67 expression (Figure 7G), and 2) potentiation of systemic immunity, evidenced by substantial elevation of key pro‐inflammatory cytokines including TNF‐α, IL‐6, and IFN‐γ (Figure 7H,I) as measured by ELISA. These complementary effects collectively underscore the treatment's capacity to not only directly inhibit tumor progression but also to elicit a coordinated anti‐tumor immune response. Our investigation revealed that DMIC‐based therapies, particularly the DMIC+L regimen, significantly altered the metabolic landscape of tumor‐infiltrating CD8^+^ T cells. A critical observation was the marked reduction in intracellular ammonia levels within these T cells following treatment (Figure 7J), demonstrating the dual functionality of our nanozyme system in both activating immune responses and mitigating AITD. Notably, while DMIC+L maintained robust T cell activation capabilities comparable to DMIC monotherapy‐likely through mechanisms involving immunogenic cell death (ICD) and direct T cell stimulation and it uniquely suppressed ammonia accumulation. This distinction is pivotal, as unchecked ammonia elevation is strongly associated with T cell exhaustion and immunotherapy resistance.^9^ The DMIC nanozyme thus emerges as a next‐generation immunomodulator that synergistically combines. The DMIC+L treatment significantly enhanced dendritic cells maturation within tumor‐draining lymph nodes, concurrently boosting both the quantity of tumor‐infiltrating CD8^+^ T cells and their granzyme B (GZMB) expression levels (**Figure** **8**A–F). This coordinated immunological response provides compelling evidence for DMIC+L's potent capacity to stimulate T cell‐mediated antitumor immunity.

In vivo evaluation demonstrated that systemic administration of DMIC+L induced superior bilateral tumor suppression compared to monotherapy regimens (DMIC, DMI+L, IC+L) in a 15‐day longitudinal study (Figure 8G). While all treatment groups exhibited delayed tumor progression relative to PBS controls (Figure 8H–N), the combination therapy achieved remarkable 85% inhibition rates for both primary and distant lesions, along with significant reductions in tumor wet weights. Notably, DMIC+L maintained a favorable safety profile with no observable weight loss or organ damage (Figures S15 and S16, Supporting Information). These findings establish DMIC‐mediated NIR‐PDT as a dual‐action therapeutic strategy that not only controls local tumor growth but also triggers systemic abscopal effects to suppress metastatic progression.

**Figure 9:**
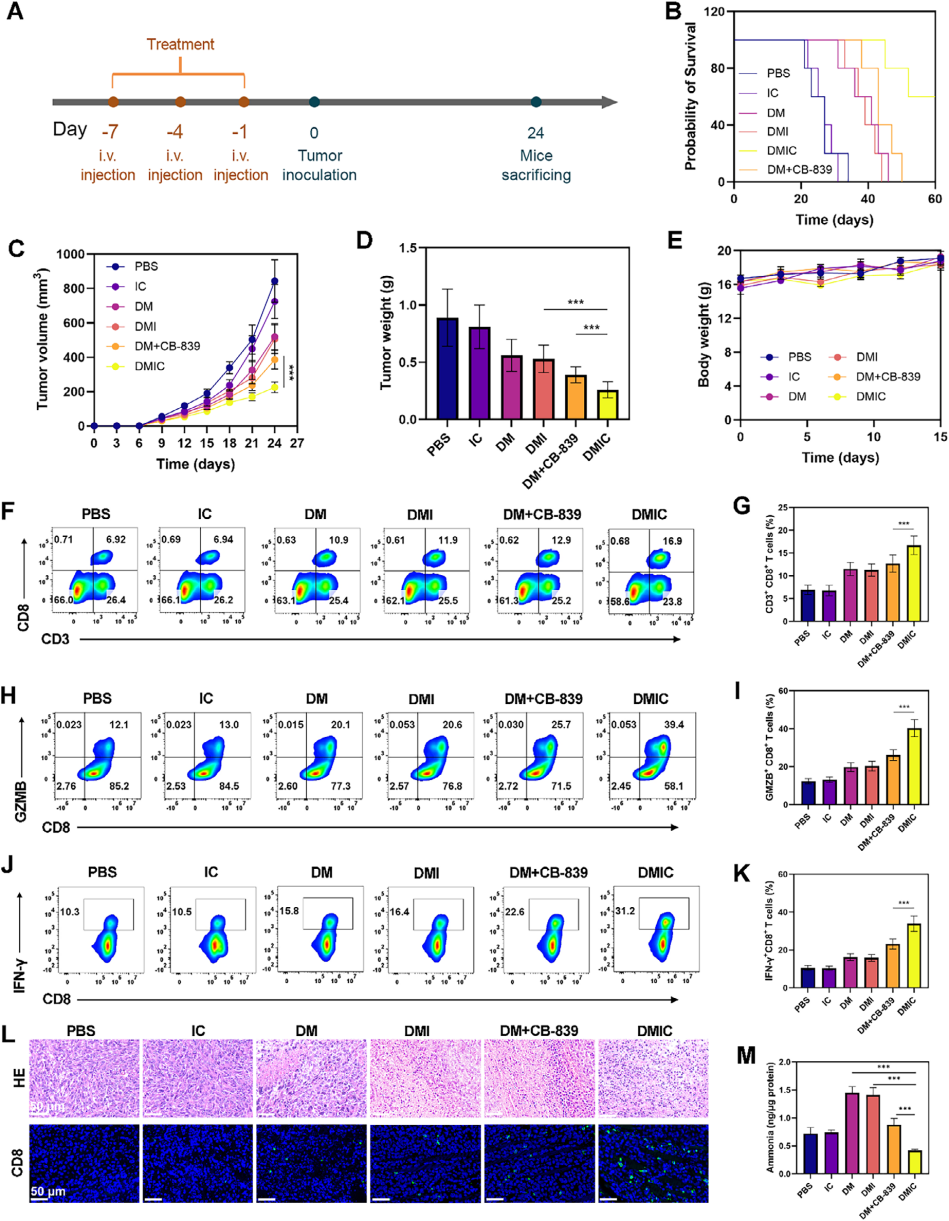
A) Schematic diagram of the timeline for the tumor prevention model. B) Survival curves after treatment. C) Tumor volumes were measured every 3 days across all groups. D) Tumor weights were recorded for each treatment at the end of the study. E) Body weights were recorded for each treatment at the end of the study of bilateral murine tumor models (*N* = 5). F) Flow cytometry data and G) quantitative analysis showing the CD3^+^ CD8^+^ T lymphocytes cells in tumors on the 24th day. H) Flow cytometry data and I) quantitative analysis showing the GZMB^+^ CD8^+^ T cells in tumors on the 24th day. J) Flow cytometry data and K) quantitative analysis showing the IFN‐γ^+^ CD8^+^ T cells in tumors. L) HE and CD8 staining of tumor sections after the indicated treatments on the 24th day. M) Ammonia levels within CD8^+^ T cells were measured 10 days after the first treatment. IR‐IHpd dose: 20 mg kg^−1^. Data are shown as the mean ± SD (*N* = 5). Statistical significance was calculated via one‐way ANOVA with Tukey's test: ^***^ *p* < 0.001.

The DMIC platform, engineered through encapsulation of BMDC membranes, demonstrates inherent T‐cell activation properties that enable tumor vaccine functionality.^[^ ^66^ ^]^ To assess this, we established a prophylactic tumor model in which Balb/c mice were administered three immunization doses prior to subcutaneous challenge with 4T1 breast cancer cells (**Figure** **9**A). While PBS and IC control groups displayed rapid tumor progression, DM, DMI, and DM+CB‐839 immunization showed transient growth inhibition (Figure 9B–D). Notably, DMIC‐vaccinated mice exhibited more substantial tumor suppression, underscoring DMIC's potential as a preventive vaccine. All experimental groups maintained consistent body weights during the study period, indicating favorable biosafety (Figure 9E). Detailed analyses demonstrated that DMIC‐treated mice possessed the highest tumor‐infiltrating T‐cell counts, along with markedly increased levels of cytotoxic marker GZMB and immunomodulatory cytokine IFN‐γ (Figure 9F–K). Histopathological examination of DMIC‐treated tumors revealed widespread necrotic areas featuring prominent nuclear condensation and fragmentation (Figure 9L), concurrent with augmented CD8^+^ T‐cell infiltration. While DM alone effectively stimulated T‐cell activation, it was unable to counteract activation‐associated ammonia buildup, leading to maximal intracellular ammonia concentrations and clear signs of AITD in this group (Figure 9M). In comparison, DMIC successfully regulated T‐cell ammonia levels and averted AITD manifestation.

## 3 Conclusion

In summary, this study presents a groundbreaking organic nanozyme system (DMIC) that synergistically integrates anthocyanin‐based NIR‐PDT, hemin‐derived peroxidase‐like catalysis, and CB‐839 to overcome AITD. Under 780 nm laser irradiation, the system generates sustained ROS through Type I/II photodynamic mechanisms and ·OH catalysis, inducing tumor immunogenic cell death while directly activating T cells. Coated with dendritic cell membranes, the DMIC efficiently targets tumor tissues, lymph nodes, and spleens, functioning as a tumor vaccine to prevent tumor recurrence. Notably, CB‐839 release mitigates intracellular ammonia toxicity in T cells, enhancing anti‐tumor immunity. This work pioneers targeted AITD inhibition by merging NIR‐PDT, metabolic engineering, and immune activation, offering a transformative paradigm for nanozyme‐based cancer immunotherapy. Future directions include optimizing nanozyme penetration in solid tumors and investigating long‐term immune memory effects.

## 4 Experimental Section

### Preparation of Tumor Cell Lysate (TCL)

The preparation of the whole TCL was modified to follow a previous research article with some modifications.^[^ ^67^ ^]^ 10^6^ 4T1 cells were collected in centrifuge tubes, washed twice with PBS, and resuspended in 1 mL RPMI‐640 culture in the base. Subsequently, the DMSO solution of IR‐IHpd was added until the final concentration was 10 µg mL^−1^, and the DMSO content was ensured to be less than 0.1%. Subsequently, the system was subjected to laser irradiation (780 nm for 10 min). Then transfer the cells to the cryopreservation tube and put it in liquid nitrogen for quick freezing, and it was taken out after 15 min and quickly put in a 37 °C water bath to thaw for 15 min. After 6 cycles of freeze‐thaw, the large organelle was removed by centrifugation (460 g, 10 min). The supernatant was collected and sterilized by 0.22 mm sterile filter to obtain TCL. Repeat the above experiment and use the hydrogen peroxide detection kit (Beyotime) to measure the content of hydrogen peroxide in tumor cells.

### Animal Tumor Models

Female BALB/c mice, aged 5–6 weeks, were obtained from Vital River Laboratories (Beijing, China). To establish the tumor model, 5 × 10⁶ 4T1 cells were subcutaneously inoculated into the right flank to form primary tumors, while 1 × 10⁶ 4T1 cells were injected into the left flank to induce distant tumors. All animal procedures were performed in accordance with the guidelines for Care and Use of Laboratory Animals of the Ministry of Health in the People's Republic of PR China and approved by the Animal Ethics Committee of Guangxi Medical University (Approval number: 2025‐KYL (025)).

### Statistical Analysis

Data analyses were conducted using the GraphPad Prism 5.0 software. For variance analysis, One‐way analysis of variance (ANOVA) with Tukey's post hoc test was used. *p*‐values of <0.05 were considered significant. ^*^ *p* < 0.05, ^**^ *p* < 0.01, ^***^ *p* < 0.001.

## Conflict of Interest

The authors declare no conflict of interest.

## Supporting information



Supporting Information

## Data Availability

The data that support the findings of this study are available from the corresponding author upon reasonable request.
