# Supplementary material for: Biomimetic Organic Nanozyme as Tumor Vaccines for Targeted Suppression of Ammonia‐Induced T Lymphocyte Death to Augment Breast Cancer Immunotherapy
Source: Adv Sci (Weinh). 2025 Nov 7;13(11):e18037. doi: 10.1002/advs.202518037 (PMC12931173; doi:10.1002/advs.202518037)
Supplement: Supplementary file 1 — Supporting Information [file ADVS-13-e18037-s001.docx]

**Supporting Information**

**Biomimetic Organic Nanozyme as Tumor Vaccines for Targeted Suppression of** **Ammonia-Induced T Lymphocyte Death to Augment Breast Cancer Immunotherapy**

*Meng Suo*, Deyi Yang, Mingpu Yang, Jue Wang, Dingfeng Zhang, Daoming Zhu*, Qingyong Xu *, Yanni Song**

Dr. M. Suo, Dr, M. Yang, Dr. J. Wang, and Dr. D. Zhang

Research Center of Nanomedicine Technology, The Second Affiliated Hospital of Guangxi Medical University, Nanning, 530000, China.

Email:

Dr. D. Yang

Department of Breast Surgery, Harbin Medical University Cancer Hospital, 150 Haping Road, Harbin, 150081 China.

Prof. Q. Xu and Prof. Y. Song

Department of Breast Surgery, Harbin Medical University Cancer Hospital, 150 Haping Road, Harbin, 150081 China**.**

Email: [xuqingyong@hrbmu.edu.cn](mailto:xuqingyong@hrbmu.edu.cn) (Q. Xu), [1525@hrbmu.edu.cn](mailto:1525@hrbmu.edu.cn) (Y. Song)

Prof. D. Zhu

Department of General Surgery, Guangdong Provincial Key Laboratory of Precision Medicine for Gastrointestinal Tumor, Nanfang Hospital, Southern Medical University, Guangzhou, Guangdong 510515, China.

Email: [zhudaoming666@smu.edu.cn](mailto:zhudaoming666@smu.edu.cn)

**Experimental Procedures**

**Materials**

DCFH-DA, DSPE-PEG2000, DSPE-Hyd-PEG2000, CB-839, 1,1'-Dioctadecyl-3,3,3',3'-Tetramethylindodicarbocyanine Perchlorate (Dil), 3,3'-Dioctadecyloxacarbocyanine Perchlorate (DiO), Dihydrorhodamine 123 (DHR123), 9,10-Anthracenediyl-bis(methylene)dimalonic acid (ABDA), Hydroxyphenyl Fluorescein (HPF) and ELISA kit used in this work were purchased from Guangzhou Ruiao Biotechnology Co., Ltd.(China). All of the aqueous solutions were prepared using purified deionized (DI) water purified with a purification system (Direct-Q3, Millipore, USA). The other solvents used in this work were purchased from Sinopharm Chemical Reagent (China) and Shanghai Macklin Biochemical Technology Co., Ltd. (China).

**Cell lines**

4T1 mouse breast cancer cell line (RRID: CVCL_0125) was purchased from Wuhan Servicebio Technology Co., Ltd on March 6, 2025.and incubated in RPMI-1640 medium supplemented with 10% FBS in a humidified atmosphere at 37℃.

**Preparation and characterization of IR-IHpd**

**Instruments**

^1^H nuclear magnetic resonance (NMR) spectra were measured on a Bruker AVANCE 300M fully digital superconducting NMR spectrometer using DMSO-*d*_6_ as solvents, and tetramethylsilane (TMS; δ = 0 ppm) was chosen as the internal reference. High-resolution mass spectra (HRMS) were obtained on a Bruker ultrafleXtreme mass spectrometer system operated in matrix-assisted laser desorption and ionization–time-of flight (MALDI-TOF) mode. UV-vis spectra and fluorescence spectra were measured at the HORIBA Duetta spectrometer.

**‌Synthesis of 5-Iodo-2,3,3-trimethyl-3H-indole (1)**

To a solution of 4-iodophenylhydrazine (511 mg, 2.18 mmol) and 3-methyl-2-butanone (320 mg, 3.72 mmol) in ethanol (20 mL), H₂SO₄ (32 μL) was added. The mixture was stirred under reflux for 4 hours. After cooling to room temperature, Na₂CO₃ was added to adjust the pH to 7.0. The solution was poured into water (20 mL) and extracted with DCM (2 × 20 mL). The combined organic phases were washed with water (2 × 20 mL), dried over anhydrous Na₂SO₄, and concentrated under reduced pressure to afford a red solid (607 mg, 97% yield). ^1^H NMR (300 MHz, CDCl_3_) δ 7.67 – 7.59 (m, 2H), 7.30 (s, 1H), 2.28 (s, 3H), 1.31 (s, 6H).

**‌Synthesis of 5-Iodo-1,2,3,3-tetramethyl-3H-indol-1-ium iodide (2)‌**

A mixture of compound ‌**1**‌ (1.05 g, 3.68 mmol) and iodomethane (0.98 mL, 7.37 mmol) in acetonitrile (20 mL) was added to a pressure tube and heated at 90 °C in an oil bath for 10 hours. After cooling to room temperature, the resulting precipitate was filtered and washed with toluene (3 × 20 mL) and diethyl ether (3 × 20 mL) to afford a brown solid (1.3 g, 85% yield). ^1^H NMR (300 MHz, DMSO-*d_6_*) δ 8.30 (d, *J* = 1.5 Hz, 1H), 8.01 (dd, *J* = 8.4, 1.6 Hz, 1H), 7.73 (d, *J* = 8.4 Hz, 1H), 3.94 (s, 3H), 2.75 (s, 3H), 1.53 (s, 6H).

**‌Synthesis of 1-(3-Hydroxypropyl)-5-iodo-2,3,3-trimethyl-3H-indol-1-ium iodide (3)‌**

A mixture of ‌compound **2**‌ (1.05 g, 3.68 mmol) and **‌**iodopropanol‌ (1.37 g, 7.37 mmol) in acetonitrile (20 mL) was added to a pressure tube and heated at 90 °C in an oil bath for 10 hours. After cooling to room temperature, the resulting precipitate was filtered and washed with toluene (3 × 20 mL) and diethyl ether (3 × 20 mL) to afford a brown solid (1.2 g, 69% yield).

**Synthesis of 2-((E)-2-((E)-6-chloro-5-(2-((E)-5-iodo-1,3,3-trimethylindolin-2-ylidene)ethylidene)cyICohex-1-en-1-yl)vinyl)-1-(3-hydroxypropyl)-5-iodo-3,3-dimethyl-3H-indol-1-ium iodide (4)‌**

A mixture of compound ‌**2‌** (427.1 mg, 1 mmol) and ‌2-Chloro-1-formyl-3-(hydroxymethyl)cyclohexene (174 mg, 1 mmol) was dissolved in 5 mL of a mixed solvent (n-butanol = 7:3, v/v) in a flask. The mixture was heated at 110 °C for 2 hours. After cooling to room temperature, compound ‌**3**‌ (471 mg, 1 mmol) was added. The reaction system was then refluxed at 120 °C for 12 hours, with water generated during the reaction removed continuously using a Dean-Stark trap. The entire process was carried out under nitrogen atmosphere. Upon completion, the solvent was removed under reduced pressure, and the residue was washed with diethyl ether. The crude product was purified by silica gel column chromatography (eluent: dichloromethane /methanol = 25:1, v/v) to afford a green solid (358 mg, 39.5% yield). ^1^H NMR (300 MHz, DMSO-*d_6_*) δ 7.80 (d, *J* = 1.6 Hz, 1H), 7.75 (dd, *J* = 8.3, 1.6 Hz, 1H), 7.62 (dd, *J* = 8.3, 1.7 Hz, 2H), 7.36 (d, *J* = 13.3 Hz, 2H), 7.22 (d, *J* = 8.4 Hz, 1H), 6.99 (d, *J* = 8.4 Hz, 2H), 5.73 (d, *J* = 13.2 Hz, 1H), 4.10 – 4.01 (m, 1H), 3.88 (s, 3H), 3.60 (s, 2H), 3.40 (s, 5H), 2.60 (s, 2H), 1.76 (d, *J* = 5.4 Hz, 4H), 1.57 (s, 12H).

**Synthesis of 1-(3-((3-(3-(2-carboxyethyl)-13,18-diethyl-2,8,12,17-tetramethylporphin-7-yl)propanoyl)oxy)propyl)-2-((E)-2-((E)-2-chloro-3-(2-((E)-5-iodo-1,3,3-trimethylindolin-2-ylidene)ethylidene)cyICohex-1-en-1-yl)vinyl)-5-iodo-3,3-dimethyl-3H-indol-1-ium iodide (IR-IHpd)‌**

A mixture of ‌iron porphyrin‌ (62 mg, 0.1 mmol), ‌compound **4**‌ (90 mg, 0.1 mmol), ‌EDCI‌ (28.7 mg, 0.15 mmol), and ‌DMAP‌ (6 mg, 0.05 mmol) was dissolved in anhydrous DMF (10 mL) and stirred at room temperature for 12 hours, with reaction progress monitored by TLC. After completion, the mixture was poured into anhydrous diethyl ether to precipitate a solid, which was filtered under reduced pressure. The crude product was purified by silica gel column chromatography (eluent: dichloromethane/methanol = 10:1, v/v) to afford a brown solid (52 mg, 34.5% yield). ^1^H NMR (300 MHz, DMSO-*d_6_*) δ 7.96 (s, 1H), 7.80 (s, 2H), 7.61 (s, 1H), 7.32 (s, 1H), 7.18 (s, 2H), 6.96 (s, 1H), 6.17 – 5.94 (m, 4H), 5.72 (s, 1H), 4.02 (s, 2H), 3.86 (s, 3H), 3.57 (s, 5H), 3.10 – 2.86 (m, 13H), 1.90 – 1.45 (m, 22H), 0.98 (d, *J* = 6.4 Hz, 6H); ^13^C NMR (151 MHz, DMSO-*d_6_*) δ 172.4, 148.5, 143.8, 143.1, 138.0, 137.6, 131.6, 127.3, 114.2, 102.7, 90.1, 60.9, 49.3, 35.2, 32.3, 26.5, 21.4, 21.0, 20.2, 19.2, 14.4; HRMS *m/z* 1381.2736 [M]^+^ (calcd for C_68_H_72_N_6_O_4_ICI_2_Fe^+^, 1381.2742).

**Computational details**

The ground-state geometries of molecules were optimized using density functional theory (DFT) method. The polarizable continuum model (PCM), with self-consistent reaction field (SCRF), was used to consider the bulky solvation effects. Time-dependent DFT (TD-DFT) method was utilized at the same level of theory to calculate energy levels of singlet, triplet states and their gap (Δ_EST_) based on the optimized singlet-state geometries. Analytical frequency calculations were also performed at the same level of theory to confirm that the optimized structures were at a minimum point. Above quantum chemical calculations were carried out by using Gaussian 16 program.

**Dendritic Cells (DCs) Antigen Presentation and Activation.**

Bone Marrow-Derived DCs (BMDCs) were isolated from the femoral and tibial bone of 6-8 week male Balb/c mice according to a previous method^1^. Briefly, mouse bone marrow cells were generated by flushing femoral and tibial bones with RPMI-1640, then erythrocytes were removed, and the remaining cells were cultured in the presence of recombinant 20 ng/mL GM-CSF and 10 ng/mL IL-4 medium for 5 days. Next day, 4T1 TIC were incubated with BMDCs for 12 h to acquire activated BMDCs. To assess the maturity rate, these DCs were stained with anti-CD80 (Biolegend) and anti-CD86 antibodies (Biolegend) and then analyzed by Flow cytometer.

**Preparation and characterization of IR-IHpd and CB-839 co-loaded liposomes (IC), BMDCs membranes (DM), DM coated IC (DMIC), IR-IHpd loaded liposomes (IL) and DM coated IL (DMI)**

The DM were prepared according to the previous work^2^. A mixture of IR-IHpd (1 mg), CB-839 (1 mg) DSPE-Hyd-PEG2000 (5 mg) and chloroform (1 mL) was sonicated (12 W output) to obtain clear solution. The mixture was quickly injected into 9 mL of water, which was sonicated vigorously in water for 2 min. The mixture was stirred in fume food for 12 h to remove the DMIC chloroform. IC suspension was performed for ultrafiltration (molecule weight cut off 100 kDa) at 3000 g for 30 min. Finally, the IC was mixed with 1mg DM and then repeatedly coextruded through 200 nm pores. The resultant DMIC were centrifuged and washed with PBS several times to remove the excess DM. IL was prepared using the same method as IC, except that CB-839 was removed. DMI was prepared using the same method as DMIC, except that IC was replaced with IL. IR-IHpd loading capacity were calculated by UV-vis spectra at the UV-vis spectrophotometry Lambda 35 (PerkinElmer). Loading capacity = M_drug_/M_DMIC_. where M refers to the mass. Protein expression was determined by western blot. The size distribution and zeta potential were measured by DLS. The morphology of synthesized materials was observed with field-emission TEM (JEM-F200).

**Detection of ROS**

ABDA, HPF, and DHR123 were employed to estimate the generation of ^1^O_2_, ·OH and total ·O_2_^−^ under NIR laser irradiation (0.5 W/cm^2^, 2 min). The concentration of IR-IHpd was 0.01 mg/mL. Typically, the DMIC was mixed with ABDA (final concentration 5 μg/mL) in PBS buffer solution (pH 7.4). Subsequently, the system was exposed to NIR laser irradiation (780 nm, 0.5 W/cm^2^, 8 min). The degradation of ABDA was calculated by the absorption decrease at 380 nm (A/A_0_). The generation of ·OH and ·O_2_^−^ was investigated by using HPF and DHR123, respectively. Added DMIC to PBS solution containing 5 μM HPF/DHR123. After irradiation with 780 nm laser (0.5 W/cm^2^) for 8 min, fluorescence emission in the range of 470-700 nm was tested under 460 nm excitation. The activation of HPF was calculated by the increase in fluorescence intensity at 515 nm, and that of DHR123 was calculated at 525 nm (I/I_0_). Electron Paramagnetic Resonance (EPR) experiments were conducted on Bruker EMXplus EPR spectrometer.

**POD-like activity of DMIC**

To examine the enzyme-like activity of the DMIC, catalytic oxidation experiments were performed using TMB substrate according to previous reports with modifications^3^. Firstly, TMB solution (10 mg/mL) and H_2_O_2_ (10 mM) was prepared in a NaAc-HAc buffer at pH 4.0. Subsequently, a solution of 10 μg DMIC was added and mixed into the reaction mixture. The absorbance at 652 nm was measured. Kinetic measurements of the nanozymes were conducted following previous reports with modifications. The maximum reaction velocity (V*_max_*) and Michaelis constant (K*_m_*) of obtained nanozymes were calculated following the Michaelis-Menten equation:

$$V=\frac{V_{max}*S}{K_{m}+S}$$

*V_max_* represented the maximal reaction velocity, S was the substrate concentration, and K*_m_* was the Michaelis constant reflecting the affinity of the nanozymes towards the substrate.

***In vitro* cancer targeting study**

4T1 cells were seeded in 24-well plates and cultured for 12 h. Then, 100 μL DiO labeled IC or DMIC (20 μg/mL IR-IHpd) was added to the medium. Then, the cells were incubated for different time at 37℃ and 5% CO_2_ and washed with PBS three times. The cells were harvested, stained with DAPI and imaged by using a fluorescence microscope (IX81, Olympus, Japan).

**Cell isolation from spleens**

Spleens were aseptically isolated from mice and incubated at 37℃ in 10% FBS RPMI media containing 1.4 mg/ml collagenase A (Roche) and 30 μg/ml DNase I for 60 min. The treated lung tissue and spleen was dissociated over the 70 μm cell strainer (Fisherbrand). Strainer was washed to collect single-cell suspension. Red blood cells were lysed with ACK lysing buffer (Lonza) for 5 min following by washing of cells with culture media. Cells were counted and adjusted to 5 × 10^6^ cells/ml. Spleen cells were washed twice in fresh magnetic-activated cell sorting (MACS) buffer. CD8^+^ T cells were magnetically purified via negative selection using MACS cell separation system according to the manufacturer’s protocols. Add CD8^+^ T cells to 1640 medium (containing 10% FBS) and adjust the cell density to 2 × 10^6^/mL, while adding 100U/mL IL-2, CD3/CD28 beads. The expression of RhCG in CD8^+^ T cells was detected by a fluorescence microscope (IX81, Olympus, Japan).

***In vitro* CD8^+^ T cells targeting study**

10^5^ CD8^+^ T cells were seeded in 24-well plates. Then, 100 μL Dil labeled IC or DMIC (20 μg/mL IR-IHpd) was added to the medium. Then, the cells were incubated for different time (0.5, 2 and 4 h) at 37℃ and 5% CO_2_ and washed with PBS three times. The cells were harvested, stained with Lyso-Tracker Green (Beyotime) and imaged by using a fluorescence microscope (IX81, Olympus, Japan).

**Hydrogen peroxide (H_2_O_2_) detection *in vitro***

4T1 cells (1 × 10^6^ per plate) were incubated with three different groups: (1) PBS+ L (780 nm, 0.5W/cm^2^, 10 min); (2) IR-IHpd+L and (3) DMIC+L. IR-IHpd concentration was 10 μg/mL. After 2 hours, the cells were collected by centrifugation (225 g, 3 min), followed by dispersion in acetone (1 mL for 5 × 10^6^ cells) and sonication for 10 min in an ice bath. Afterward, the suspension was centrifuged (8000 g, 10 min) to collect the supernatant. The H_2_O_2_ content was assessed by the commercial H_2_O_2_ assay kit (Beyotime) based on the product protocol.

**Intracellular ROS, anti-cancer effect and immunogenic cell death (ICD) detection.**

4T1 cells (1.5 × 10^5^ per well) were seeded in a 12-well plate for 12 h. Then the cells were incubated and treated with 8 different groups: (1) PBS; (2) L (780 nm, 0.5W/cm^2^, 10 min); (3) DMI; (4) IC; (5) DMI+L; (6) DMIC; (7) IC+L; (8) DMIC+L. IR-IHpd concentration was 10 μg/mL. The cells were harvested, stained with HPF and DAPI and imaged by using a fluorescence microscope (IX81, Olympus, Japan).

For anti-cancer effect detection, 4T1 cells were incubated in six-well plates at 37℃ with 5% CO_2_ for 24 h; afterward, the culture medium was replaced by new culture medium, cells were incubated with 8 different groups as mentioned above. After incubation for another 6 h. The viability of 4T1 cells was determined by a CCK-8 cell cytotoxicity assay. Subsequently, we used a similar method to evaluate the killing effect of different concentrations of IR-IHpd on 4T1 cells.

For ICD detection, 4T1 cells (1.5 × 10^5^ per well) were seeded in a 12-well plate for 12 h. Then the cells were incubated and treated with 6 different groups: (1) PBS; (2) L (780 nm, 0.5W/cm^2^, 10 min); (3) DMI+L; (4) DMIC; (5) IC+L; (6) DMIC+L. IR-IHpd concentration was 10 μg/mL. The cells were washed with PBS three times, fixed with 4% PFA and permeabilized with 0.1% Triton X-100 for 10 min. After washing with PBS three times, the cells were blocked with 10% FBS, and incubated with Anti-Calreticulin antibody or Anti-HMGB1 antibody (Bioss) and fluorescent labeled secondary antibody for 30 min. The cells were washed with PBS three times, then stained with DAPI for 20 min. Finally, the cells were washed with PBS three times and observed using CLSM. Fluorescence intensity was measured by ImageJ software. For quantification of released HMGB1 and ATP in medium, the medium was collected after the cells were treated with materials. Then 20 μL medium was used for ELISA detection or ATP Assay Kit.

**Transcriptome gene sequencing**

4T1 breast tumor cells were seeded in 6-well plates (1 × 10^6^ cells/well) and cultured overnight. Cells were treated by PBS or DMIC+L as in the apoptosis experiment described above. The total RNA was extracted from cells using the TRIzol. Later, the 150 bp double ended sequencing was performed using Illumina Hiseq instrument. All differentially expressed genes between groups were shown in volcano graphs, and specific DEGs were represented by heat maps. Moreover, screening and identification of differentially expressed mRNA for KEGG and GO pathway analysis was carried out.

**Transwell experiment on Bone marrow-derived dendritic cells (BMDCs) stimulation in vitro**

BMDCs were isolated from 8-week-old BALB/c mice bone marrow. For BMDCs maturation assay, 1 × 10^5^ 4T1 cells were treated by the above six groups and then cocultured with 1 × 10^6^ BMDCs in the transwell culture system, and BMDCs were then isolated by anti-CD11c magnetic beads (Thermo Fisher). Then BMDCs were stained with FITC-anti-CD80 and PE-anti-CD86 (Abcam). Finally, the cells were sorted using flow cytometer (Beckman-Coulter, USA). The secretion levels of cytokines including TNF-α, IL-6, and IFN-γ in the samples were tested with ELISA kits.

**T Cells Response Measurements**

BMDCs were generated from bone mesenchymal stem cells harvested from mice and were preseeded in 6-well plates at a density of 1×10^5^ per well and incubated for 24 h. The 4T1 cells were treated with IR-IHpd+L and then the dead tumor cells were incubated with BMDCs for 24 h. IR-IHpd concentration was 0.1mg/mL. Spleens were surgically removed to prepare single-cell suspensions as mentioned above, which were then added into the plates. After incubation for 24 h, CB-839 or IC or DMIC (CB-839 concentration was 1μM) were then added into the plates. After incubation for another 8 days, the upper layer of the medium was added to the plates pre-seeded with 4T1 tumor cells (3×10^4^ per well in 24-well plates), and the concentration of LDH in the supernatant were detected by LDH Assay Kit.

An Ammonia Assay Kit (Abcam) was used to quantify ammonia. Briefly, cells were collected and lysed on ice and equal numbers (approximately 1 × 10^6^) of CD8^+^ T cells from different groups were used for ammonia extraction. Additionally, we utilized one-tenth of the cell lysate to measure the protein level. The ammonia levels in whole-cell lysate or subcellular organelles were detected according to the manufacturer’s protocols.

***In vivo* biodistribution study**

Female BALB/c mice aged 5-6 week were purchased from Vital River Company (Beijing, China). BALB/c mice were subcutaneously injected with 5 × 10^6^ 4T1 cells into the right flank. When tumors reached 300 mm^3^, tumor bearing mice (n = 3) received an intravenous (*i.v.*) injection of 100 μL PBS containing DiR or DiO or Dil labeled IC or DMIC (with a IR-IHpd dose of 10 mg/kg). Then the mice were sacrificed at different time after injection to collect the tumors, LNs and major organs for fluorescence imaging by *In Vivo* Imaging System (IVIS). Lymph nodes and tumor tissues from each group were collected for immunofluorescence analysis.

***In vivo* anti-tumor study**

Female BALB/c aged 5-6 week were purchased from Vital River Company (Beijing, China). BALB/c mice were subcutaneously injected with 5 × 10^6^ 4T1 cells into the right flank. When the tumor grows to approximately 100 mm^3^, treatment is carried out on day 0. The mice were firstly divided randomly into different groups (Each group includes 5 mice): (1) PBS; (2) L (780 nm, 0.5W/cm^2^, 10 min); (3) DMI+L; (4) DMIC; (5)IC+L; (6) DMIC+L. The IR-IHpd dose was 10 mg/kg. Nanoparticles were administered intravenously and undergo phototherapy on day 0 and 3. Mice body weight and tumor volume in all groups were monitored every 3 days. A caliper was employed to measure the tumor length and tumor width, and the tumor volume was calculated according to following formula. Tumor volume = tumor length × tumor width^2^ / 2. On the 15th day, mice were sacrificed (Due to ethical limitations, the mice were sacrificed at this time). Five main organs (heart, liver, spleen, lung, and kidney) of all mice were harvested, washed with PBS, and fixed with paraformaldehyde for histology analysis. And the tumor tissues were weighed, and fixed in 4% neutral buffered formalin, processed routinely into paraffin, and sectioned at 4 μm. The primary tumor sections were stained with HE, TUNEL, Ki-67 and finally examined by using fluorescence microscope (IX81, Olympus, Japan). Fluorescence intensity was measured by ImageJ software. On the 15th day, Tumor infiltrating CD8^+^ T cells were isolated using a CD8^+^ T cell sorting kit (Thermo Fisher) and their ammonia content were measured as mentioned above. The inguinal lymph nodes (LN) were harvested. The frequency of DC maturation in the LNs was then examined by CD11c^+^ cell sorting kit (Thermo Fisher) and flow cytometry after immunofluorescence staining with FITC-anti-CD80 and APC-anti-CD86 (Biolegend)**.** To study the T cells content and function, LNs were harvested from mice in different groups and treated with flow cytometry after immunofluorescence staining with FITC-anti-CD3 and PE-anti-CD8 (Abcam); FITC-anti-CD8 and APC-anti-GZMB (Biolegend). To analysis treatment-induced cytokine secretion, whole blood was collected from mice at 3 days post first treatment. The serum concentration of proinflammatory cytokines including TNF-α, IL-6 and IFN-γ were then analyzed with ELISA kits (Neobioscience Co., Ltd., China) according to the manufacturer's instructions. Another group of mice repeated the experiment, and their survival was observed for 60 days.

**Bilateral tumor experiment**

Female Balb/c mice aged 5-6 week were purchased from Vital River Company (Beijing, China). Balb/c mice were subcutaneously injected with 5 × 10^6^ 4T1 cells into the right flank (primary tumors) and 1 × 10^6^ 4T1 cells into the left flank (distant tumors), respectively. The mice were first divided randomly into 6 different groups and treated as mentioned above. Mice body weight and tumor volume in all groups were monitored every 3 days. A caliper was employed to measure the tumor length and tumor width, and the tumor volume was calculated according to following formula. Tumor volume = tumor length × tumor width^2^ / 2. On the 15th day, mice were sacrificed (Due to ethical limitations, the mice were sacrificed at this time). The distant tumor tissues were weighed, and fixed in 4% neutral buffered formalin, processed routinely into paraffin, and sectioned at 4 μm. Then the tumor sections were stained with HE and anti-CD8 antibodies and fluorescence labeled secondary antibody and finally examined by using fluorescence microscope (IX81, Olympus, Japan).

**Tumor prevention experiment**

Female Balb/c aged 5-6 week were purchased from Vital River Company (Beijing, China). The mice were treated with 6 different groups: (1) PBS; (2) IC; (3) DMI; (4) DM+CB-839; (5) DM and (6) DMIC. The treatment was conducted once a day on the day -6, -3 and 0. The IR-IHpd dose was 20 mg/kg. After 6 days of the first treatment, the mice were subcutaneous injected with 5 × 10^6^ 4T1 cells into the right flank. Mice body weight and tumor volume in all groups were monitored every 3 days. A caliper was employed to measure the tumor length and tumor width, and the tumor volume was calculated according to following formula. Tumor volume = tumor length × tumor width^2^/ 2. On the 24th day, mice were sacrificed (Due to ethical limitations, the mice were sacrificed at this time). The tumor tissues were weighed, and fixed in 4% neutral buffered formalin, processed routinely into paraffin, and sectioned at 4 μm. The tumor sections were stained with hematoxylin-eosin (HE) staining and anti-CD8 antibodies and fluorescence labeled secondary antibody and finally examined by using fluorescence microscope (IX81, Olympus, Japan). On the 24th day, the live CD8^+^ T lymphocytes were isolated from tumors, and the subpopulations of T cells were finally analyzed on a flow cytometer as mentioned above.

**References**

(1) Chen, H.; Luo, X.; Huang, Q.; Liu, Z.; Lyu, M.; Chen, D.; Mo, J.; Zhu, D. Platelet membrane fusion liposome loaded with type I AIE photosensitizer to induce chemoresistance cancer pyroptosis and immunogenic cell death for enhancing cancer immunotherapy. *Chemical Engineering Journal* **2023**, *476*, 146276. DOI: 10.1016/j.cej.2023.146276.

(2) Ma, X.; Kuang, L.; Yin, Y.; Tang, L.; Zhang, Y.; Fan, Q.; Wang, B.; Dong, Z.; Wang, W.; Yin, T.; Wang, Y. Tumor–Antigen Activated Dendritic Cell Membrane-Coated Biomimetic Nanoparticles with Orchestrating Immune Responses Promote Therapeutic Efficacy against Glioma. *ACS nano* **2023**, *17* (3), 2341-2355. DOI: 10.1021/acsnano.2c09033.

(3) Zhu, D.; Chen, H.; Huang, C.; Li, G.; Wang, X.; Jiang, W.; Fan, K. H_2_O_2_ Self-Producing Single-Atom Nanozyme Hydrogels as Light-Controlled Oxidative Stress Amplifier for Enhanced Synergistic Therapy by Transforming “Cold” Tumors. *Advanced Functional Materials* **2022**, *32* (16), 2110268. DOI: 10.1002/adfm.202110268.

**Supplementary figures**

**Figure S1**. ^1^H NMR spectrum (300 MHz, CDIC_3_, 298 K) of compound **1**.

**Figure S2**. ^1^H NMR spectrum (300 MHz, DMSO-*d_6_*, 298 K) of compound **2**.

**Figure S3**. ^1^H NMR spectrum (300 MHz, DMSO-*d_6_*, 298 K) of compound **4**.

**Figure S4**. ^1^H NMR spectrum (300 MHz, DMSO-*d_6_*, 298 K) of compound **IR-IHpd**.

**Figure S5**. **^13^**C NMR spectrum (150 MHz, DMSO-*d_6_*_,_ 298K) of compound **IR-IHpd.**


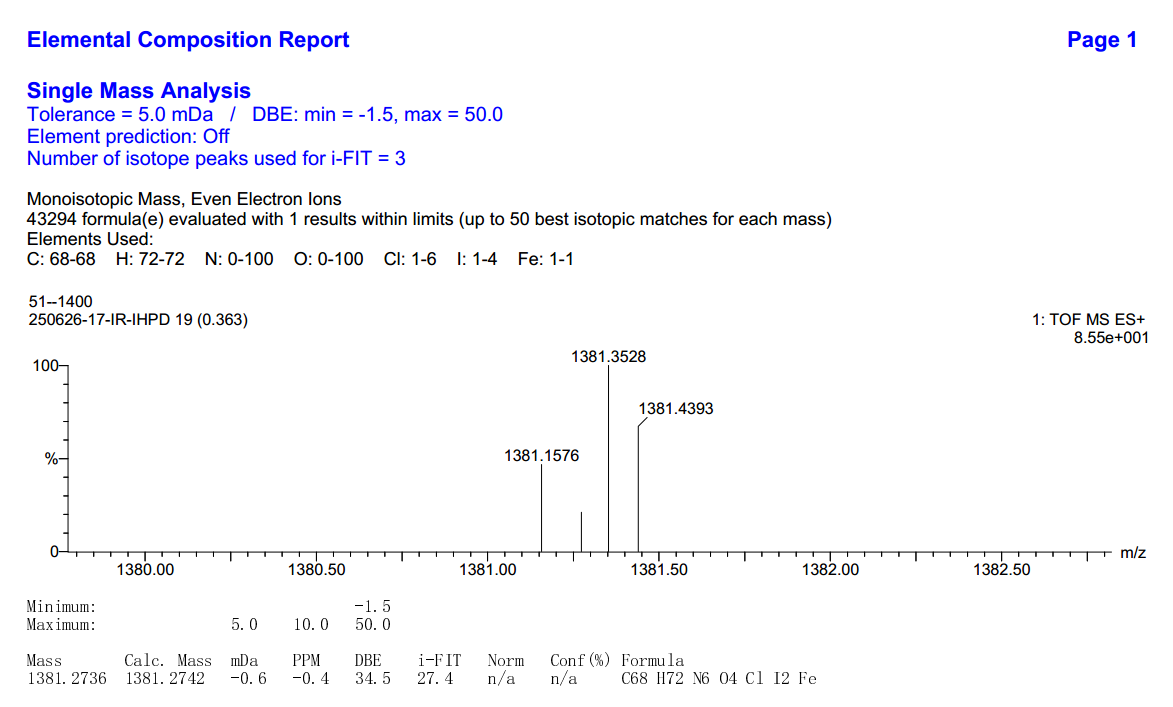


**Figure S6**. HRMS of compound **IR-IHpd.**


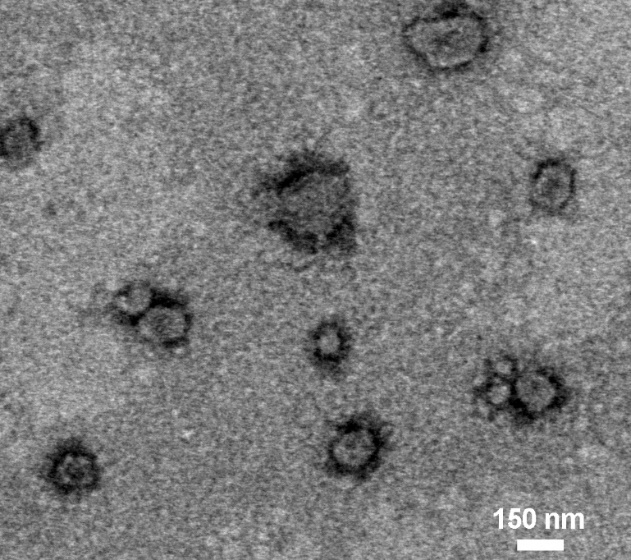


**Figure S7.** TEM image of DM.


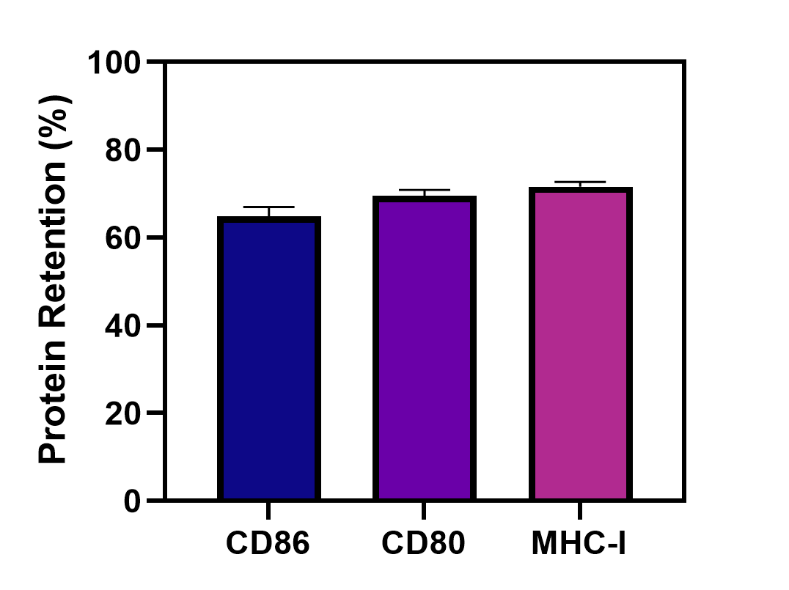


**Figure S8.** The key membrane protein retention on DMIC through ImageJ software (n=3).


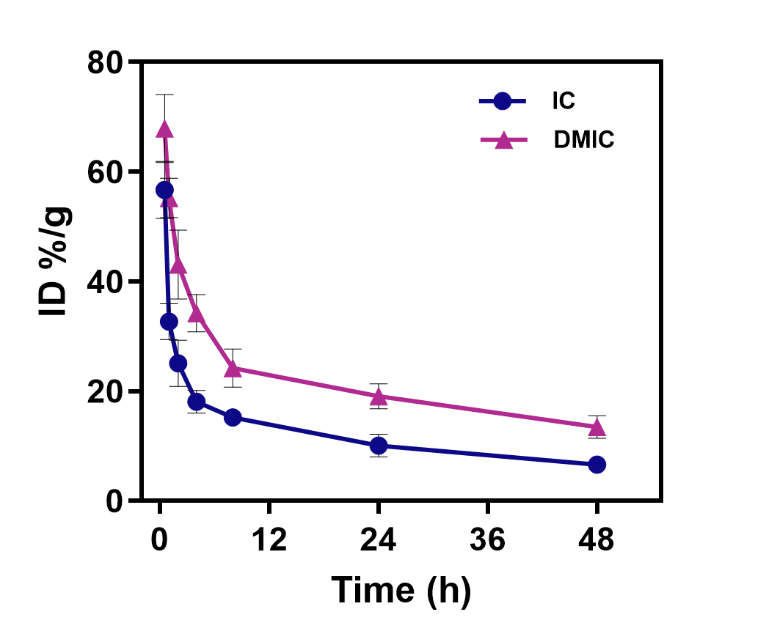


**Figure S9**. Pharmacokinetic curves of IC and DMIC (n=3).


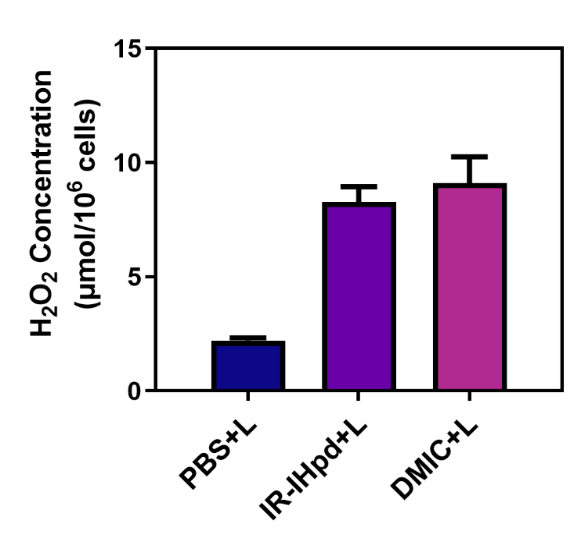


**Figure S10.** Production of intracellular H_2_O_2_ by indicated treatment (n=3).


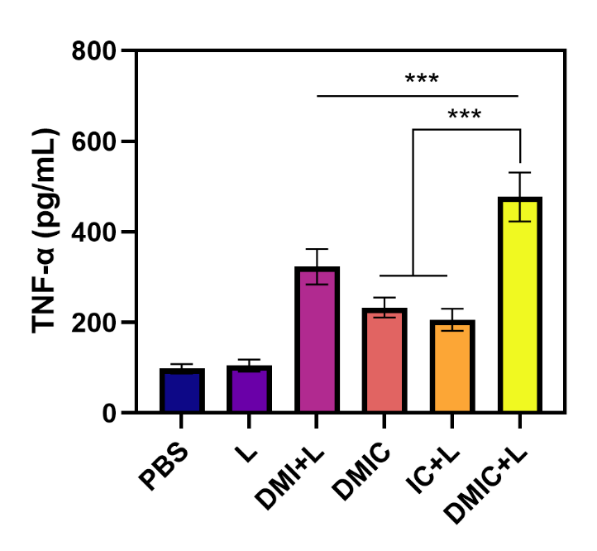


**Figure S11**. ELISA analysis of the levels of TNF-⍺ in BMDCs culture medium after different treatments (n=3). Statistical significance was calculated via one-way ANOVA with Tukey’s test: ***p < 0.001.


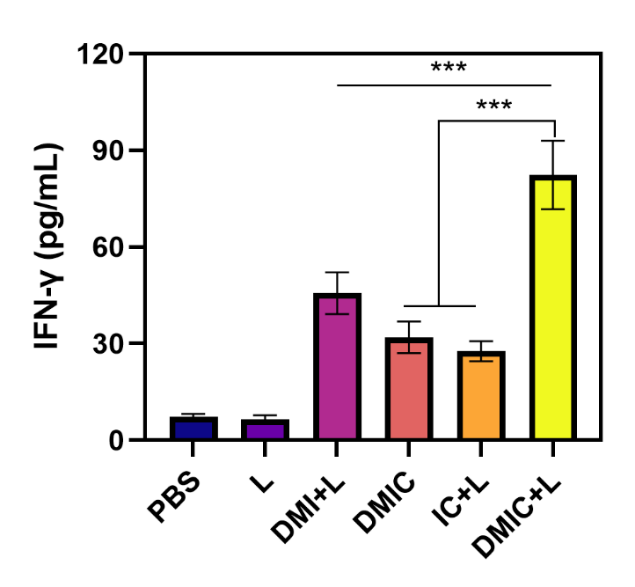


**Figure S12**. ELISA analysis of the levels of IFN-γ in BMDCs culture medium after different treatments (n=3). Statistical significance was calculated via one-way ANOVA with Tukey’s test: ***p < 0.001.


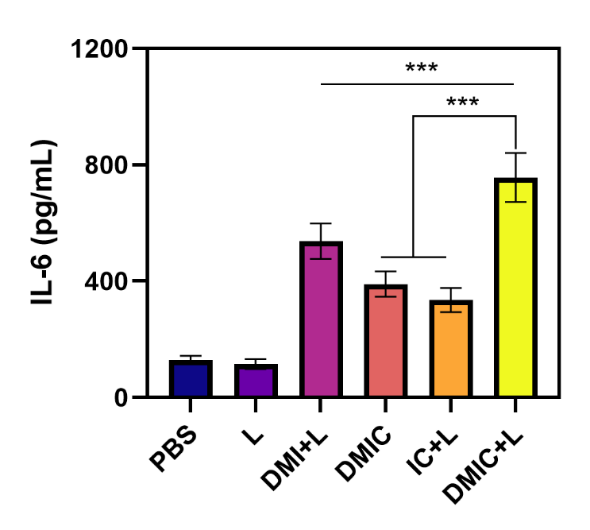


**Figure S13**. ELISA analysis of the levels of IL-6 in BMDCs culture medium after different treatments (n=3). Statistical significance was calculated via one-way ANOVA with Tukey’s test: ***p < 0.001.


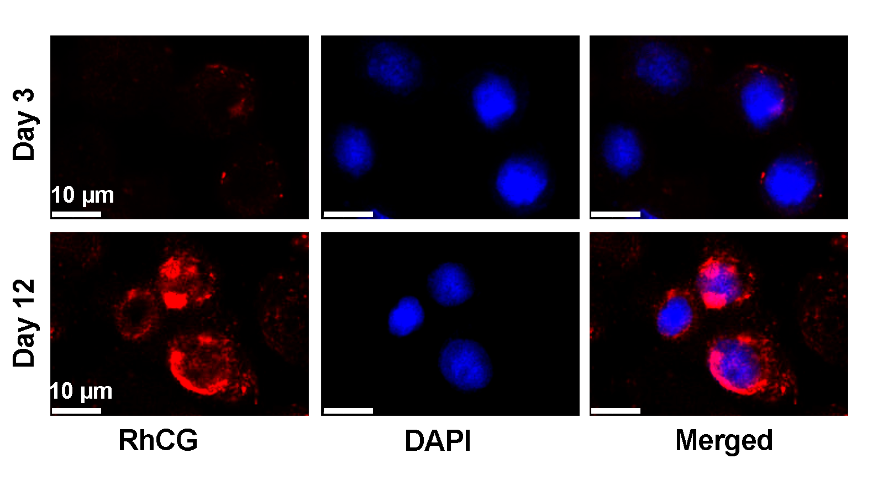


**Figure S14.** Immunofluorescence staining of RhCG in splenic CD8^+^ T cells at different time points after activation.


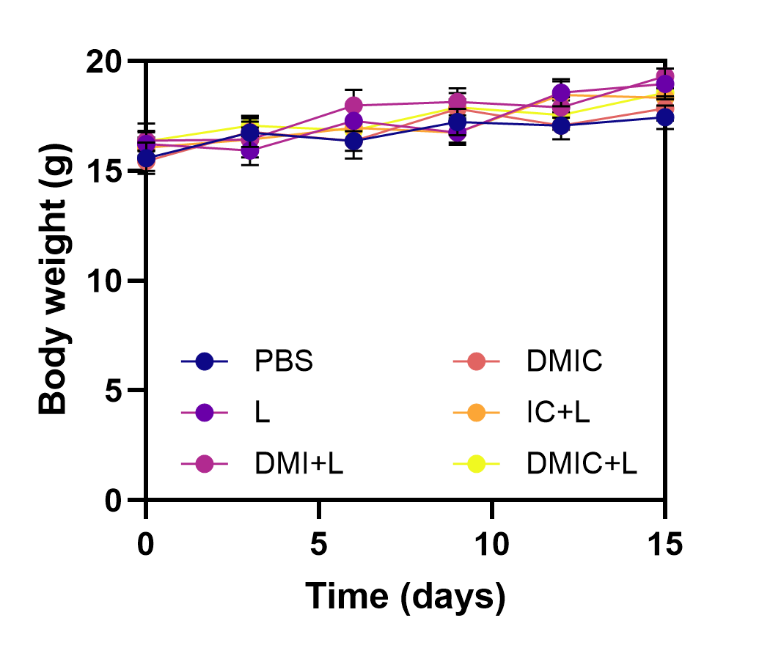


**Figure S15**. Body weights were recorded for each treatment at the end of the study of bilateral murine tumor models (n=5). Statistical significance was calculated via one-way ANOVA with Tukey’s test: ***p < 0.001.


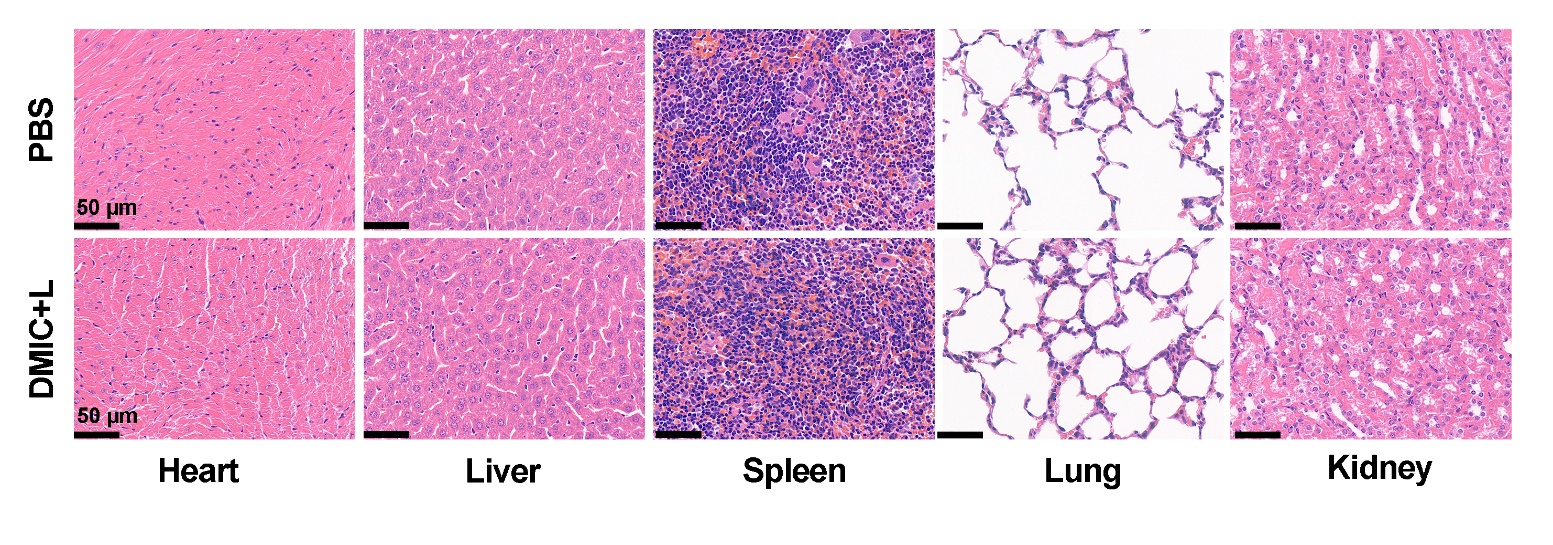


**Figure S16**. HE-stained images for the mice's major organs, including the heart, lung, liver, kidneys, and spleen from mice after different treatments.
